# Supplementary material for: Carbon redirection via tunable Fenton-like reactions under nanoconfinement toward sustainable water treatment
Source: Nat Commun. 2024 Apr 1;15:2808. doi: 10.1038/s41467-024-47269-6 (PMC10985074; doi:10.1038/s41467-024-47269-6)
Supplement: Supplementary file 1 — Supplementary Information [file 41467_2024_47269_MOESM1_ESM.pdf]

Supplementary Information

for

Carbon Redirection via Tunable Fenton-like Reactions Under Nanoconfinement Toward Sustainable Water Treatment

Xiang Gao[1#], Zhichao Yang[1,2#], Wen Zhang[3], Bingcai Pan[1,2*]

[1]State Key Laboratory of Pollution Control and Resources Reuse, Nanjing University, Nanjing 210023 China

[2]Research Center for Environmental Nanotechnology (ReCENT), School of Environment, Nanjing University, Nanjing 210023 China

[3]John A. Reif, Jr. Department of Civil and Environmental Engineering, New Jersey Institute of Technology, 323 Martin Luther King Blvd., Newark, NJ, USA

[#] Both authors contribute equally to this work.

[*]Corresponding author. E-mail: bcpan@nju.edu.cn

The supplementary files contain 15 methods, 1 note, 47 figures, and 6 tables.

Outline

Supplementary Methods...5
Supplementary Method 1. Analysis of the products on the catalysts by MALDI-TOF-MS and GPC. 5
Supplementary Method 2. Preparation of catalysts...5
Supplementary Method 3. DFT calculation 7
Supplementary Method 4. Reusability of the catalysts. 8
Supplementary Method 5. Pre-oxidation of the catalysts...8
Supplementary Method 6. Calculation of the average pH in the nanopores based on Donnan theory-based model. 9
Supplementary Method 7. MD and FEM simulation. 10
Supplementary Method 8. Diffusion of the reactants and products in the nanopores. 11
Supplementary Method 9. Materials and chemicals. 12
Supplementary Method 10. Determination of PMS concentration. 13
Supplementary Method 11. EPR characterization. 13
Supplementary Method 12. UHPLC-MS/MS analysis. 14
Supplementary Method 13. Screening and quantification of oxidation products in UHPLC-MS/MS analysis...15
Supplementary Method 14. Ion chromatography (IC) measurement. 15
Supplementary Method 15. Extraction of oxidation products on the residual ACNTs. 16
Supplementary Notes...17
Supplementary Note 1. Definition of conversion, yield and selectivity. 17
Supplementary Tables...18
Supplementary Table 1. Information of the main products of PhOH oxidation from UHPLC-MS/MS analysis...18
Supplementary Table 2. The enthalpy of reaction of PhOH and representative oligomers...23
Supplementary Table 3. The diffusion coefficients of the reactants and products. 24
Supplementary Table 4. Characteristics of the wastewater secondary effluents. 25
Supplementary Table 5. UHPLC operational conditions for different organic compounds. 26
Supplementary Table 6. Operational conditions of UHPLC-MS/MS analysis. 27
Supplementary Figures...28
Supplementary Fig. 1 TEM images of ACNTs at different scale bars. 28
Supplementary Fig. 2 XRD spectra of three ACNTs. 29
Supplementary Fig. 3 TEM images of Mn_3O_4@nACNT. 30
Supplementary Fig. 4 Mn 3*s* XPS spectra of Mn_3O_4@*n*ACNT and bulk Mn_3O_4 NPs. 31
Supplementary Fig. 5 Raman spectra of Mn_3O_4@*n*ACNT and bulk Mn_3O_4 NPs. 32
Supplementary Fig. 6 Conversion of PhOH in the absence of either PMS or catalysts. 33
Supplementary Fig. 7 Conversion of PhOH in *n*ACNT/PMS systems. 34
Supplementary Fig. 8 Effect of Mn^{2+} leaching on the conversion of PhOH. 35
Supplementary Fig. 9 Effect of pre-equilibration of the catalysts and PMS on PhOH conversion in different oxidation systems. 36
Supplementary Fig. 10 Effect of pre-equilibration of the catalysts and PhOH on PhOH conversion in different oxidation systems. 37

Supplementary Fig. 11 The UHPLC-MS/MS spectra of oligomers and hydroxylated products detected in the oxidation processes. 38
Supplementary Fig. 12 The UHPLC-MS/MS spectra of organic acids and carbonyl compounds detected in the oxidation processes. 39
Supplementary Fig. 13 The ratio of products adsorbed on the catalysts to the total products generated. The error bars represent the standard deviations from triplicate tests. 40
Supplementary Fig. 14 Effect of BQ and HQ on PhOH conversion and oligomer yield in bulk Mn_3O_4/PMS and Mn_3O_4@20ACNT/PMS. 41
Supplementary Fig. 15 Effect of BQ concentration on PhOH conversion in bulk Mn_3O_4/PMS. ...42
Supplementary Fig. 16 Reaction selectivity towards oligomers at different PhOH conversions in four oxidation systems. 43
Supplementary Fig. 17 The average degree of oligomerization at different PhOH conversions in four oxidation systems. 44
Supplementary Fig. 18 Yields of the $C_{7\text{-}11}$ degradation products at different PhOH conversions in four oxidation systems. 45
Supplementary Fig. 19 GPC spectra of the products extracted from the reacted catalysts. The green shadow indicates the region of eluted polymers. 46
Supplementary Fig. 20 MALDI-TOF-MS spectra of products adsorbed on the catalysts in Mn_3O_4@55ACNT/PMS and Mn_3O_4@120ACNT/PMS....47
Supplementary Fig. 21 Characterization of Mn_3O_4/*n*ACNT and PhOH conversion in Mn_3O_4/nACNT/PMS. 48
Supplementary Fig. 22 Consumption of PMS in different oxidation systems. 49
Supplementary Fig. 23 The reusability of the catalysts. 50
Supplementary Fig. 24 Effect of PMS concentration on (a) the kinetics of PhOH degradation and (b) the oligomer yield in different oxidation systems. 51
Supplementary Fig. 25 Effect of pH on PhOH conversion in (a) Mn_3O_4/PMS, (b) Mn_3O_4@120ACNT/PMS, (c) Mn_3O_4@55ACNT/PMS, and (d) Mn_3O_4@20ACNT/PMS. 52
Supplementary Fig. 26 Plots of the net surface charge of (a) Mn_3O_4 NPs, (b) Mn_3O_4@120ACNT, (c) Mn_3O_4@55ACNT, and (d) Mn_3O_4@20ACNT versus the solution pH values. 53
Supplementary Fig. 27 Removal of PhOH by different catalysts in the absence of PMS at pH 3.0 and 5.0....54
Supplementary Fig. 28 Kinetics of PhOH conversion in the secondary effluent of (a) textile dyeing and (b) chemical industry wastewater treatment processes....55
Supplementary Fig. 29 Isosurface map of the electron spin density of the radicals generated from oxidation of bisphenol A, 4-chlorophenol, and aniline. 56
Supplementary Fig. 30 TEMP-trapped EPR spectra of different oxidation systems. 57
Supplementary Fig. 31 Effect of solvents on the conversion of PhOH in different oxidation systems. 58
Supplementary Fig. 32 (a) The concentration of dissolved SO_4^{2-} after oxidation of PhOH by pre-oxidized catalysts. (b) Adsorption of SO_4^{2-} by different catalysts. 59
Supplementary Fig. 33 Degradation of PMSO and formation of $PMSO_2$ in different oxidation systems. 60
Supplementary Fig. 34 The kinetic isotope effect for PhOH conversion in (a) Mn_3O_4/PMS and (b) Mn_3O_4@20ACNT/PMS. 61

Supplementary Fig. 35 Effect of O_2 concentration on PhOH conversion and oligomer yield in (a–b) Mn_3O_4/PMS and (c–d) Mn_3O_4@20ACNT/PMS....62
Supplementary Fig. 36 Effect of $NaNO_3$ on PhOH oxidation by pre-oxidized catalysts....63
Supplementary Fig. 37....64
Supplementary Fig. 38 Formation of 4-chlorophenol at 20% PhOH conversion after adding Cl^-. 65
Supplementary Fig. 39 Effect of Cl^- on quinone formation (a–d) and conversion of PhOH (e–h) in different oxidation systems....66
Supplementary Fig. 40 Oxidation of BQ and product selectivity in (a) Mn_3O_4/PMS and (b) Mn_3O_4@20ACNT/PMS....67
Supplementary Fig. 41 Oxidation of HQ and product selectivity in (a) Mn_3O_4/PMS and (b) Mn_3O_4@20ACNT/PMS....68
Supplementary Fig. 42 Effect of the concentration of PhOH on the kinetics of PhOH conversion in different catalytic oxidation systems....69
Supplementary Fig. 43 MD simulations of $PhO^•$-involved reactions under nanoconfinement with different spatial sizes. (a) A snapshot of the established simulation models. (b) Effective collisions for $PhO^•$ coupling in different oxidation systems....70
Supplementary Fig. 44 Overview of FEM simulations. (a) Top view, (b) front view, and (c) 3D view of the hollow tubes....71
Supplementary Fig. 45 The computed concentration distribution of (a) $PhO^•$ and (b) dimer in the mid-section of a 5-nm tube at 50% PhOH conversion....72
Supplementary Fig. 46 Plots of computed ratio of oligomers to organic acids versus the experimental results....73
Supplementary Fig. 47 Scheme for the product analysis....74
Supplementary References....75

Supplementary Methods

Supplementary Method 1. Analysis of the products on the catalysts by MALDI-TOF-MS and GPC.

The presence of adsorbed products of highly polymerization was verified using matrix-assisted laser desorption/ionization time-of-flight mass spectrometry (MALDI-TOF MS; Atouflex Speed, Bruker Inc., USA). In brief, using dichloromethane as the eluent, the reacted catalysts were subjected to Soxhlet extraction at 410 K for 24 h. Subsequently, the obtained solutions were evaporated and dried at 333 K. Part of the obtained pale-yellow solid residues were re-dissolved in 1.0 mL THF and filtered through 0.22 μm PTFE filters. The resulting solution was thoroughly mixed with α-cyano-4-hydroxycinnamic acid, which was used the matrix for MALDI-TOF-MS analysis (337 nm laser). The spectra were recorded in negative mode.

The average molecular weights of the extracted products were analyzed using a gel permeation chromatograph (GPC; Waters) equipped with microstyragel columns and an RI 2414 detector at 308 K. The pale-yellow solid residues were dissolved in 2.0 mL THF and filtered through 0.22 μm PTFE filters. THF was used as the eluent with a flow rate of 1.0 mL min^{-1}. The molecular weights were calibrated against monodispersed polystyrene standards.

Supplementary Method 2. Preparation of catalysts.

Synthesis of nanoconfinement templates. The AAO membranes with different pore sizes were initially dried at 378 K for three hours to remove the adsorbed water. Next, the AAO membranes were placed between two polished quartz plates and annealed for 4 h at 1053 K to prevent curling during the subsequent chemical vapor deposition (CVD) process. In the CVD experiments, the annealed AAO membranes were placed upright in a custom quartz boat located in the middle of a single-stage tubular furnace. The reactor temperature increased to 1023 K at a rate of 1.0 K min^{-1} under Ar flow (40 mL min^{-1}). Once the temperature stabilized, 2.0 mL min^{-1} of ethylene gas was fed into the tubular furnace to deposit carbon films inside the pores of AAO membranes. After 2 h,

the ethylene gas was turned off, and the furnace temperature was lowered to room temperature at a rate of 1.0 K min^{-1} to obtain nanoconfinement templates with different pore diameters, i.e., *n*ACNTs confined inside AAO channels (*n* = 20, 55, and 120 representing the pore diameters of ACNTs).

Loading Mn_3O_4 on the outer surface of ACNT. The carbon-deposited AAO templates were stirred in 5.0 M NaOH solutions at 323 K for 12 h to completely dissolve the AAO templates. The obtained ACNTs were sonicated with 40 mL concentrated HNO_3 for 2 h to generate acidic groups on the outer surface. The resulting solids were immersed in 0.07 M $KMnO_4$ solutions. The suspension was then transferred to Teflon-lined reactors and ethanol (1:4, $V_{ethanol}/V_{solution}$) was added to fill with the reactors. The mixtures were autoclaved at 433 K for 4 h. The resulting suspension contained the Mn_3O_4/*n*ACNT nanocomposites and the unloaded Mn_3O_4 NPs. The desired Mn_3O_4/*n*ACNT nanocomposites were separated from the suspension based on the density differences. In brief, after static precipitation, the suspension was carefully poured off and ultrapure water was added to re-disperse the precipitates. After a number of cycles, the obtained black Mn_3O_4/*n*ACNT nanocomposites were then vacuum-dried at 313 K for 18 h and stored in a vacuum oven at 298 K before use.

Synthesis of confined catalysts. Mn_3O_4 NPs were grown inside the pores of the carbon-deposited AAO templates using the hydrothermal method. Specifically, potassium permanganate ($KMnO_4$) was used as the manganese precursor in aqueous solutions. To ensure the similar load of Mn_3O_4 NPs in different samples, the concentration of $KMnO_4$ was set as 0.07 M, 0.06 M, 0.05 M, and 0.04 M for loading in the pores of 20 nm, 55 nm, 120 nm, and bulk phase, respectively. The carbon-deposited AAO templates were immersed in corresponding aqueous solutions and sonicated for 20 min. Subsequently, they were transferred to Teflon-lined reactors separately and ethanol (1:4, $V_{ethanol}/V_{solution}$) was finally added to fill with the reactors. The mixtures were autoclaved at 433 K for 4 h.

After reaction, the hydrothermally treated materials were taken out and placed in 1.0

mM H_2SO_4 and sonicated for 10 s. Afterwards, the surfaces of Mn_3O_4-loaded materials were wiped by ethanol to remove the NPs outside the pores. After that, the materials were stirred in 5.0 M NaOH aqueous solutions at 323 K for 12 h to completely dissolve the AAO templates. The mixtures were subjected to ball milling to truncate the Mn_3O_4-loaded *n*ACNTs (~60 μm in length). Ball milling was conducted on an AGO-2S centrifugal planetary ball mill at a speed of 300 r min^{-1}. Furthermore, the samples were filtered and washed by ultrapure water until the pH of the filtrates was neutral. The materials were then vacuum-dried at 313 K for 18 h and stored in a vacuum oven at 298 K before use. The obtained catalysts were named Mn_3O_4@*n*ACNTs, where *n* = 20, 55, 120 representing the pore size.

Supplementary Method 3. DFT calculation

DFT calculation. The geometry optimization and the electronic energy calculation were conducted in the gas phase on Gaussian 16 software at M06-2X/6-311G* level with dispersion corrections (the DFT-D3 method). The collision diameters of the compounds of concern are calculated by Chemcraft software. $C_{12}H_{10}O_2$, $C_{18}H_{14}O_3$, and $C_{24}H_{18}O_4$ were selected to represent the dimer, trimer, and tetramer. The enthalpy of formation (Δ_fH) was calculated based on the following reaction: $6x\text{C} + x\text{O} + 4x\text{H} \rightarrow C_{6x}H_{4x+2}O_x$, where x (1, 2, 3, 4) represents the number of rings in the oligomers. Δ_fH of oligomers at 298 K is therefore calculated as:

$$\Delta_f H(0\text{K}) = \sum_{\text{atoms}} (\Delta_f H(atom, 0\text{K})) - \sum D_0 = \sum_{\text{atoms}} (\Delta_f H(atom, 0\text{K})) - (\sum_{\text{atoms}} E(atom) - E - E_{\text{ZPE}}) \quad (1)$$

$$\begin{aligned}\Delta_f H(298\text{K}) &= \Delta_f H(0\text{K}) + H_\text{m}(298\text{K}) - H_\text{m}(0\text{K}) - \sum_{\text{atoms}} (H_\text{m}(atom, 298\text{K}) - H_\text{m}(atom, 0\text{K})) \\ &= \sum_{\text{atoms}} (\Delta_f H(atom, 0\text{K})) - \sum_{\text{atoms}} E(atom) + (E + H_{\text{corr}}) - \\ &\quad \sum_{\text{atoms}} (H_\text{m}(atom, 298\text{K}) - H_\text{m}(atom, 0\text{K})) \\ &= 6x\Delta_f H_\text{m}(\text{C}, 0\text{K}) + (4x+2)\Delta_f H_\text{m}(\text{C}, 0\text{K}) + x\Delta_f H_\text{m}(\text{C}, 0\text{K}) - \\ &\quad (6xE(\text{C}, 0\text{K}) + (4x+2)E(\text{H}, 0\text{K}) + xE(\text{O}, 0\text{K})) + (E + H_{\text{corr}}) - \\ &\quad -6x(H_\text{m}(\text{C}, 298\text{K}) - H_\text{m}(\text{C}, 0\text{K})) - (4x+2)(H_\text{m}(\text{C}, 298\text{K}) - \\ &\quad H_\text{m}(\text{C}, 0\text{K})) - x(H_\text{m}(\text{O}, 298\text{K}) - H_\text{m}(\text{O}, 0\text{K}))\end{aligned} \quad (2)$$

where Δ_fH_m (atom, 0 K) is the enthalpy of formation of the elements (C, H, O) at 0 K, and (H_m (atom, 298 K)-H_m (atom, 0 K)) represents the enthalpy correction to the

elements (C, H, O); these two parameters are obtained from the reference [1]. E (atom, 0 K) is the electronic energy of the elements (C, H, O) at 0 K; E represents the electronic energy of oligomers at 298 K; H_{corr} is the thermal correction to enthalpy.

Calculation of the spin distribution in radicals. The geometry of the target radical was optimized on Gaussian 16 software at B3LYP/6-311G* level. The density-based solvation model was applied to simulate the aqueous environment. Single point calculations were performed at the CCSD(T)-Def2-TZVPP level. Electron spin densities were calculated and used to quantify the degree of unpaired spins at different sites in the radical.

Supplementary Method 4. Reusability of the catalysts.

Briefly, 0.02 g of the catalyst was mixed with 266.7 mL of 200 μM PhOH. The reaction was initiated by adding 2.0 mM PMS. After the reaction, the catalysts were collected by suction filtration, and were lyophilized after rinsing with ultrapure water for three times. The resulting solids were subjected to the next run. To offset the loss of catalysts during operation, the volume of reaction solution was gradually decreased according to the mass of the obtained solids. After five continuous runs, the residual catalysts were extracted following the procedure detailed in Supplementary Method 1. The extracted catalysts were subjected to another run following the same procedure described above.

Supplementary Method 5. Pre-oxidation of the catalysts.

Typically, 8.0 mg of catalyst was added to a 10-mL centrifuge tube, followed by adding 8.0 mL of PMS solution (25 mM, pH = 7.0). After 60-min reaction, the mixture was carefully centrifuged, and the catalyst solids were washed several times with ultrapure water until PMS in the supernatant cannot be detected by the KI method [2]. Then, the pre-oxidized catalyst solids were collected via centrifugation.

The as-obtained pre-oxidized catalysts were added to a 20-mL solution containing 50 μM PhOH (pH = 7.0) and allowed to react for 60 min under magnetic stirring at 293.2 ± 0.3 K. During the reaction, the suspension was sampled at specific intervals,

and immediately filtered through 0.22 μm PTFE membranes for analysis of the concentrations of PhOH and sulfate ions.

Supplementary Method 6. Calculation of the average pH in the nanopores based on Donnan theory-based model.

According to the results obtained from potentiometric titration, it can be deduced that the confined Mn_3O_4 NPs carries negative charges at neutral pH, rendering them attracting hydrogen ions. The surface charge density of these confined Mn_3O_4 NPs can be converted into volume charge density (mol m^{-3}), denoted as X, by

$$X = \frac{[(R+d)^2 - R^2] \times \rho_c \times Q_H}{R^2 \times n} \quad (3)$$

where R represents the inner diameter of the ACNT pores (m); d denotes the thickness of the ACNT walls (m); ρ_c signifies the lattice density of amorphous carbon (g m^{-3}); Q_H stands for the net surface electrostatic charge acquired through potentiometric titration (mol g^{-1}), and n represents the mass fraction of Mn_3O_4 nanoparticles within the overall material composition. In the nanopores, the volume charge density results from the equilibrium of ion charge density, which is determined by the difference between the concentrations of cations (c_+) and anions (c_-):

$$X = c_+ - c_- \quad (4)$$

The concentrations of cations and anions within the pore are related to the salt concentration outside the pore and the Donnan potential at the solution-pore interface. This relationship can be expressed as:

$$c_+ = c_{s^+} \times \exp(-\Delta\varphi_D) \quad (5)$$

$$c_- = c_{s^-} \times \exp(\Delta\varphi_D) \quad (6)$$

where c_s^+ and c_s^- represent the salt concentration of the solution outside the nanoscale pore, and $\Delta\varphi_D$ corresponds to the Donnan potential at the solution-pore interface. Specifically, in our example, due to the negatively charged pore surface, $\Delta\varphi_D$ is negative. Therefore, the proton concentration within the nanoscale pore can be calculated based on the Donnan potential as follows:

$$c_{H^+} = c_{H^+,s} \times \exp(-\Delta\varphi_D) \quad (7)$$

where c_{H^+} and $c_{H^+,s}$ are the proton concentrations of the pore and the bulk solution, respectively.

Supplementary Method 7. MD and FEM simulation.

MD simulation. A box with dimensions of 8.5 nm × 7.4 nm × *h* nm (*h* = 20, 55, 120 nm) was established on the PACKMOL software [3]. The model consists of two sp^2 carbon slabs for the spatial nanoconfinement, where 2000 water molecules and 100 phenoxy radicals are randomly placed, with the spacing between any two molecules greater than 0.2 nm. MD simulations were performed on the LAMMPS molecular dynamics code with canonical (NVT) ensembles and periodic boundary conditions in all directions [4]. The ReaxFF reactive force field was included to predict the reactions of $PhO^•$ [5,6]. The parameters for ReaxFF MD simulations were derived from the previous work describing the reactive cross-linking of polymers [7]. The simulation temperature is set as 2000 K to facilitate collision within a limited simulation interval. The time step for all MD simulations was taken as 0.25 ps. A total of 6×10^6 MD steps (i.e., 1.5 ns) were taken and the simulation system reaches an equilibration after 0.5 ns. The threshold of bond order cutoff was 0.35.

FEM simulation. The hollow tubes with a length of 1.5 μm, a wall thickness of 7.0 nm, and an inner diameter of 20, 55, 120, 1000 nm, respectively, were built to represent Mn_3O_4@*n*ACNT and Mn_3O_4 NPs (Supplementary Fig. 44a–c). Note that Mn_3O_4 NPs were represented by a hollow tube with 1000 nm inner diameter to maintain the robustness of modeling and improve the comparability of results. Five reactions were defined to simplify the complex model (Supplementary Fig. 44d–g): adsorption and desorption equilibrium of PhOH on the internal surface, transformation of the adsorbed PhOH to $PhO^•$, adsorption and desorption equilibrium of $PhO^•$, dimerization of $PhO^•$ on the surface or bulk solution (reaction of adsorbed $PhO^•$ and free $PhO^•$; coupling of free $PhO^•$), and further oxidation of $PhO^•$ on the surface to quinone. 'Transport of Diluted Species', 'Chemistry', and 'Surface Reactions' modules were used to define

the mass transport and the above chemical reactions. The effective diffusion coefficient of each compound used in FEM simulations was calculated following the procedures shown in Supplementary Method 8 and detailed in Supplementary Table 3. The kinetics of PhOH degradation and the ratio of oligomers yield to organic acids yield at 50% PhOH conversion under 20-nm nanoconfinement were used as the baseline to fit the unknown equilibrium coefficients and rate constants of chemical reactions. These parameters were fixed during modeling, except for the rate constant of inner-sphere electron transfer (adsorbed PhOH to adsorbed $PhO^{\bullet}$), which was used to represent the different reactivity of Mn_3O_4 NPs under varied spatial nanoconfinement and therefore was adjusted to fit the kinetics of PhOH conversion in different oxidation systems.

Supplementary Method 8. Diffusion of the reactants and products in the nanopores.

The mean free paths (λ) of the reactants and products (i.e, PhOH, $PhO^{\bullet}$, dimer ($C_{12}H_{10}O_2$), and BQ) can be estimated according to the kinetic theory of gases.

$$\lambda = \frac{kT}{\sqrt{2}\pi d^2 p} \tag{8}$$

where k is the Boltzmann constant (1.38×10^{-23} J K^{-1}); d is the collision diameter of the target molecule, and p is the pressure. The Knudsen number (K_n) is calculated using Equation 9 to estimate the significance of Knudsen diffusion in the pores [8].

$$K_{\mathrm{n}} = \frac{\lambda}{d_{\mathrm{pore}}} = \frac{kT}{\sqrt{2}\pi d^2 d_{\mathrm{pore}} p} \tag{9}$$

The collision diameters of PhOH, $PhO^{\bullet}$, dimer, and BQ are calculated to be 0.57 nm, 0.57 nm, 0.80 nm, and 0.57 nm, respectively, based on the optimized geometries (Supplementary Method 3). Accordingly, K_n of these compounds was calculated to be 0.12–1.66 in the pores of 20–120 nm (Supplementary Table 3) and 0.01–0.03 in the pores of 1000 nm. Note that the mean free paths of the compounds would be much shorter in water than in gas. Assuming the mean free paths equal to the collision diameters of the concerned compounds, K_n in the pores of 20–55 nm (0.01–0.04) was

also higher than 0.01, indicating the contribution of Knudsen diffusion to mass transfer. Lower K_n was obtained in the 120-nm and 1000-nm pores, suggesting the absence of Knudsen diffusion. For comparison, all the effective diffusion coefficients (D_{eff}) were calculated by the Bosanquet formula [9].

$$\frac{1}{D_{\text{eff}}} = \frac{1}{D_{\text{bulk}}} + \frac{1}{D_{\text{Knudsen}}} \tag{10}$$

where D_{bulk} and $D_{Knudsen}$ are the coefficients of molecular diffusion and Knudsen diffusion, respectively. D_{bulk} was calculated according to the Wilke-Chang equation and $D_{Knudsen}$ was calculated by Equation 11 [9].

$$D_{\text{Knudsen}} = \frac{d_{\text{pore}}}{3}\sqrt{\frac{8RT}{\pi M}} \tag{11}$$

where R is the gas constant, 8.314 J K^{-1} mol^{-1}; M is the molar mass of the target. The values are detailed in Supplementary Table 3.

Supplementary Method 9. Materials and chemicals.

The AAO membranes (double-pass, 60 μm thickness, 25 mm diameter) with different pore diameters were purchased from Hefei Pu-Yuan Nano Technology Co., Ltd. (China). Peroxymonosulfate (PMS, $2KHSO_5 \cdot KHSO_4 \cdot K_2SO_4$, $KHSO_5$ > 43%), phenol (99%), benzoquinone (BQ, 98%), hydroquinone (HQ, 99%), catechol (99%), resorcinol (99%), deuterium oxide (D_2O, 99.9 atom% D), C_6D_5OH (99 atom% D), C_6D_5OD (99 atom% D), 5,5-dimethyl-1-pyrroline-N-oxide (DMPO, 98%), methyl phenyl sulfoxide (PMSO, 97%), methyl phenyl sulfone ($PMSO_2$, 98%), 2,2,6,6-tetramethylpiperidine (TEMP, 99%), dimethyl sulfoxide (DMSO, 99.9%) and acetonitrile (ACN, 99.9%) of HPLC grade were purchased from Sigma-Aldrich. Mn_3O_4, formic acid (98%), oxalic acid (99%), propanoic acid (99.5%), formaldehyde (37%), acetaldehyde (99.5%), potassium iodide (KI, 99%), sodium sulfate (Na_2SO_4, 99%), methanol (MeOH, 99.9%), *tert*-butanol (TBA, 99.5%), ethanol (99.8%), tetrahydrofuran (THF, 99.5%) and *p*-toluenesulfonyl hydrazine (TSH, 98%) were purchased from Aladdin. MnO, Mn_2O_3, MnO_2, sodium bicarbonate ($NaHCO_3$, 99.5%),

sodium carbonate (Na_2CO_3, 99.9%), acetic acid (99.9%), butyric acid (99.5%), succinic acid (99.5%), acrylic acid (99%), maleic acid (99%), fumaric acid (98%), muconic acid (97%), benzoic acid (99%), *p*-hydroxybenzoic acid (PHBA, 99.5%), succinaldehyde (97%), glutaraldehyde (50%), 4,4'-biphenol (99%) and 4-phenoxyphenol (99%) were purchased from Macklin. Sodium hydroxide (NaOH, 96%), nitric acid (HNO_3, 65%), hydrochloric acid (HCl, 38%), sulfuric acid (H_2SO_4, 98%), sodium thiosulfate ($Na_2S_2O_3$, 98.5%), sodium sulfite (Na_2SO_3, 97%), sodium nitrite ($NaNO_2$, 99%) and potassium permanganate ($KMnO_4$, 99.5%) were purchased from Shanghai Chemical Reagent Co., China. 4-(4-Phenoxyphenoxy) phenol (95%) and 4-[4-(4-phenoxyphenoxy) phenoxy] phenol (95%) were purchased from Shenzhen Atomax Co., China. All materials were used as received. Ultrapure water (18.25 MΩ cm) was used to prepare the solutions unless otherwise stated.

Supplementary Method 10. Determination of PMS concentration.

The concentration of PMS was analyzed using the KI method [2]. The suspension sampled from the reactors was immediately filtered through a 0.22-μm PTFE filter. Then, 0.25 mL of the filtrate was quickly added to 2.75 mL mixture of KI (600 mM), $NaHCO_3$ (60 mM), and phosphate buffer (50 mM, pH = 6.0), and then was diluted to 5.0 mL. After 5-min incubation, the absorbance of the solution was analyzed on a UV-visible absorption spectrophotometer at 352 nm. The concentration of PMS was calculated based on the calibration curve.

Supplementary Method 11. EPR characterization.

The reactions for detecting the reactive oxidants were carried out in 5-ml glass vials (Conditions: catalysts = 75 mg L^{-1}, PMS = 2.0 mM, pH = 7.0±0.1). After stirring for 2 min, 200 μL of the reaction solutions were sampled and immediately mixed with 20 μL 1.0-M spin trap (DMPO or TEMP). Subsequently, the mixed solution was transferred to a capillary tube, which was placed in a quartz EPR tube (φ5 × 250 mm) for EPR analysis. All spectra were obtained under the following conditions: center field = 3520 G; scan width = 150 g; scanning time = 41.943 s; microwave frequency = 9.895 GHz;

microwave power = 6.437 mW.

The reactions for detecting the phenoxy radicals were carried out in 5-ml glass vials (Conditions: catalysts = 300 mg L^{-1}, PMS = 40 mM, PhOH = 40 mM, DMPO = 100 mM, pH = 7.0±0.1). After stirring for 30 min, 200 μL of the reaction solutions were collected to determine the EPR spectra. The other operational procedures and instrumental parameters were consistent with those for detecting reactive oxidants.

Supplementary Method 12. UHPLC-MS/MS analysis.

An ultrahigh performance liquid chromatography system (UHPLC; Dionex Ultimate 3000) coupled with a Q Exactive focus Orbitrap mass spectrometer (Thermo Fisher Scientific, Bremen, Germany) was utilized to analyze the oxidation products. The Hypersil GOLD C18 column (100 mm × 2.1 mm × 1.9 μm, Thermo Fisher Scientific) was used for chromatographic separation. The MS/MS system was equipped with an electron spray ionization (ESI) source operating in negative ESI mode (ESI^-) for analysis of oxidation products like oligomers and BQ/HQ, and positive ESI mode (ESI^+) for ring-opened aldehydes and ketones. All data were acquired using a data-dependent acquisition (DDA) mode for both target and non-target analysis. In this mode, a full MS1 scan (m/z 50 – 7000) was acquired with a resolution of 70000 (at m/z 200), followed by top three MS_2 scans with a resolution of 17500 (at m/z 200) for each cycle.

The samples containing oxidation products were analyzed within 24 hours after sampling and were stored at 277 K prior to injection into the UHPLC-MS/MS system. For analysis of ring-opened aldehydes and ketones, pre-derivatization with toluenesulfonyl hydrazine (TSH) was conducted prior to injection, following the previous method. In brief, the sample, 2.0 mM hydrochloric acid, and 0.10 mM TSH (from a 50-mM TSH standard solution dissolved in acetonitrile) were allowed to react for 10 min and then subjected to UHPLC-MS/MS analysis within two hours. Detailed conditions for UHPLC-MS/MS analysis are detailed in Supplementary Table 6.

Supplementary Method 13. Screening and quantification of oxidation products in UHPLC-MS/MS analysis.

The UHPLC-MS/MS data was processed using the MS-DIAL software (version 4.70) to extract the peak positions and molecular weights of potential oxidation products. For aldehydes and ketones, the peak positions and molecular weights of the adducts with TSH were extracted by inputting the signature TSH fragment ion into the MS/MS fragment searcher pane in MS-DIAL software. Afterward, the Xcalibur Qual Browser software was used to calculate the molecular formula of organic molecules in the corresponding peaks (with element composition restriction of $C_{0-100}H_{0-200}O_{0-20}N_{0-5}S_{0-5}$). Finally, the structure of organic molecules in the corresponding peaks was predicted using the MS/MS spectra.

The accurate concentration of oxidation products with commercially available standards were quantified based on UV absorbance or signals from mass spectrometer. For those oxidation products without references, structurally similar molecules were used as references and their concentration was semi-quantified using mass spectrometer. This is a widely adopted semi-quantitative method in untargeted mass spectrometry analysis to estimate the concentration of substances without references [10-12]. For example, the C–C coupled dimers, C–O coupled dimers, trimers and tetramers of PhOH used 4,4'-biphenol, 4-phenoxyphenol, 4-(4-phenoxyphenoxy) phenol and 4-[4-(4-phenoxyphenoxy) phenoxy] phenol as the references, respectively; the ring-opened aldehydes/ketones used acetaldehyde as the reference.

Supplementary Method 14. Ion chromatography (IC) measurement.

Ion chromatography (IC, Thermo, Aquion-1100) equipped with an IonPac AS23-HC analytical column (4 mm×250 mm), a DS6 conductivity detector, and an ASRS-300 anion-regeneration suppressor was employed to measure the concentrations of small organic acids. An aqueous solution consisting of 3.2 mM Na_2CO_3 and 1.0 mM $NaHCO_3$ was used as the mobile phase with a flow rate of 0.6 mL min^{-1}. The sample injection volume was 20 μL.

Supplementary Method 15. Extraction of oxidation products on the residual ACNTs.

The acid-digested catalysts were extracted by 30-mL organic solvents to analyze the possible oxidation products adsorbed on ACNTs. Organic solvents with different polarity, including methanol, ethanol, dichloromethane, tetrahydrofuran and toluene, were used as the extraction agents. The catalysts after acid-digestion were mixed with organic solvents and stirred for 12 h. Then, the filtrates were analyzed by UHPLC-MS/MS.

Supplementary Notes

Supplementary Note 1. Definition of conversion, yield and selectivity.

The conversion of PhOH, and the yield and selectivity of oxidation products are defined as follows:

$$Conversion(\%) = \frac{C_0 - C_t}{C_0} \times 100 \quad (12)$$

$$Yield(\%) = \frac{C_p}{C_0} \times 100 \quad (13)$$

$$Selectivity(\%) = \frac{C_p}{C_0 - C_t} \times 100 \quad (14)$$

where C_0 represents the initial concentration of C atoms in PhOH, and C_t and C_p represent the C concentration of PhOH and the target product at a certain reaction time, *t*, respectively.

Supplementary Tables

Supplementary Table 1. Information of the main products of PhOH oxidation from UHPLC-MS/MS analysis.

Molecular formula	m/z	Retention time (min)	Proposed structure
		Oligomers	
$C_{12}H_{10}O_2$	185.0601	15.16	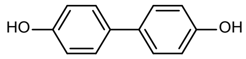
$C_{12}H_{10}O_2$	185.0601	18.82	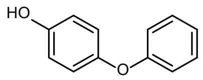
$C_{12}H_8O_2$	184.0526	15.15	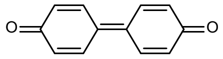
$C_{12}H_{10}O_3$	201.0549	11.43	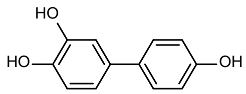
$C_{12}H_{10}O_3$	201.0549	14.97	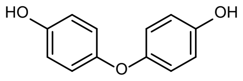
$C_{12}H_8O_3$	199.0395	14.98	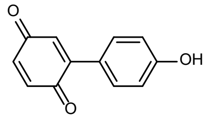
$C_{12}H_8O_3$	200.0478	16.35	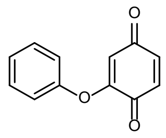
$C_{12}H_{10}O_4$	217.0494	10.98	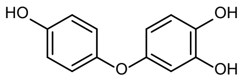
$C_{12}H_8O_4$	215.0351	15.23	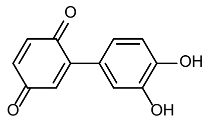
$C_{12}H_8O_4$	215.0351	16.58	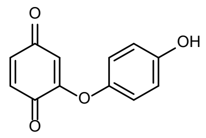
$C_{12}H_6O_4$	213.0184	20.22	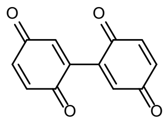
$C_{12}H_8O_5$	231.0287	13.34	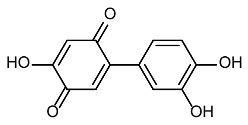

$C_{18}H_{14}O_3$	277.0856	16.61	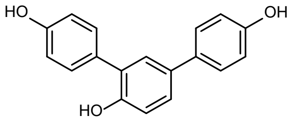
$C_{18}H_{14}O_3$	277.0856	17.39	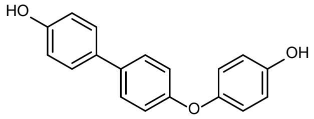
$C_{18}H_{14}O_3$	277.0856	20.97	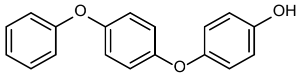
$C_{18}H_{12}O_3$	275.0714	14.72	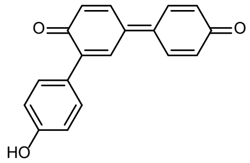
$C_{18}H_{12}O_4$	291.0652	18.07	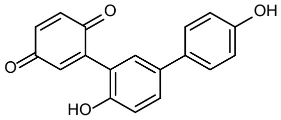
$C_{18}H_{12}O_4$	291.0658	18.72	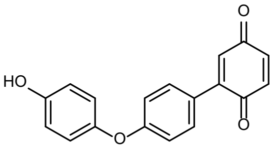
$C_{18}H_{12}O_4$	291.0652	20.86	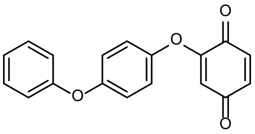
$C_{18}H_{12}O_5$	307.0602	16.75	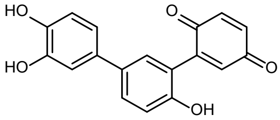
$C_{18}H_{12}O_5$	307.0602	19.86	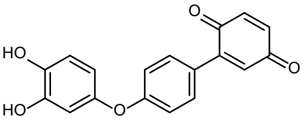
$C_{18}H_{12}O_5$	307.0602	20.78	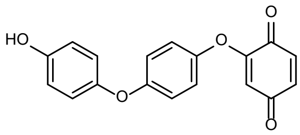
$C_{24}H_{18}O_4$	369.1118	15.15	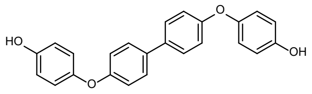
$C_{24}H_{18}O_4$	369.1118	20.92	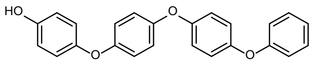
	**Quinones**		
$C_6H_4O_2$	108.0206	7.91	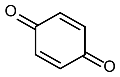

$C_6H_6O_2$	109.0284	8.53	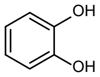
$C_6H_6O_2$	109.0284	4.34	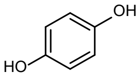
$C_6H_6O_2$	109.0284	7.76	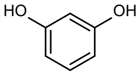
$C_6H_4O_3$	123.0084	3.89, 4.78, 9.12	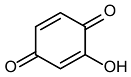
$C_6H_6O_3$	125.0243	7.89, 11.82	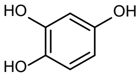
**Organic acids**			
$C_6H_6O_4$	141.0182	6.00	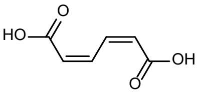
$C_6H_6O_5$	157.0136	4.13	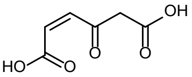
$C_5H_6O_2$	97.0293	3.32	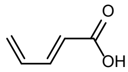
$C_5H_6O_3$	113.0242	3.20	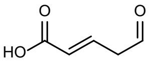
$C_5H_8O_3$	115.0387	3.70	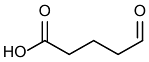
$C_5H_6O_4$	129.0191	2.61, 3.54	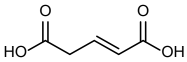
$C_5H_4O_4$	127.0032	1.84	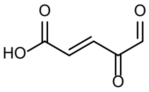
$C_5H_{10}O_6$	165.0402	4.07	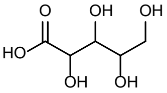
$C_4H_4O_3$	99.0086	1.77, 3.07	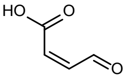
$C_4H_4O_4$	115.0026	4.46	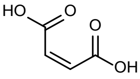
$C_4H_4O_4$	115.0026	2.85	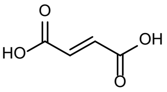
$C_4H_6O_4$	117.0182	3.31	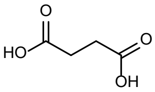

$C_4H_8O_5$	135.0298	9.50	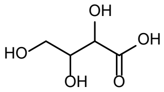
$C_4H_6O_6$	149.0089	4.04	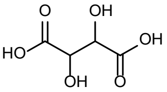
$C_3H_4O_4$	103.0026	2.18	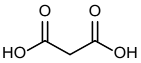
$C_3H_6O_4$	105.0194	8.33	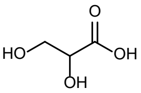
$C_3H_4O_2$	71.0127	2.62	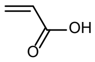
$C_3H_6O_3$	89.0243	1.09, 2.16, 4.85	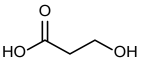
$C_3H_6O_2$	74.0362	8.05*	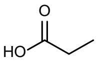
$C_2H_4O_2$	60.0206	7.92*	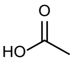
$C_2H_2O_3$	73.9998	7.14*	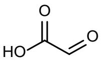
$C_2H_2O_4$	89.9948	43.75*	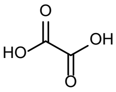
CH_2O_2	42.0049	8.77*	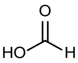

Aliphatic carbonyl compounds

**Molecular formula**	**Monoisotopic mass**	**m/z**	**Retention time (min)**	**Proposed derivatized structure**
$C_6H_6O_2$	110.0362	279.0492	18.80	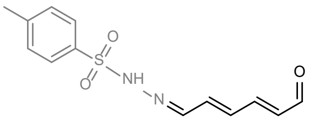
$C_6H_8O_3$	128.0468	296.0764	18.55	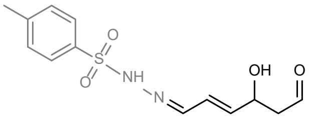
$C_5H_{10}O$	86.0726	255.1151	14.88	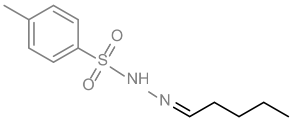
$C_5H_8O_2$	100.0519	269.0954	12.59	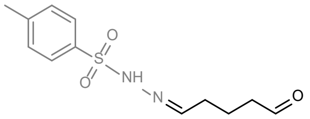

C_4H_8O	72.0570	241.099	13.61	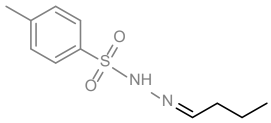
$C_4H_4O_2$	84.0206	253.0641	11.99	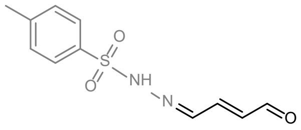
C_3H_6O	58.0413	227.0834	12.62	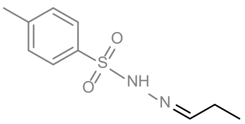
C_2H_4O	44.0257	213.0681	11.98	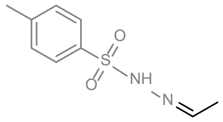
$C_2H_2O_2$	58.0049	395.0842	14.77	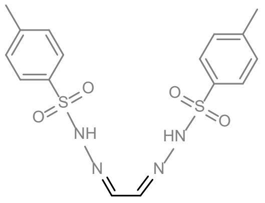
$C_2H_4O_2$	60.0206	229.0742	9.71	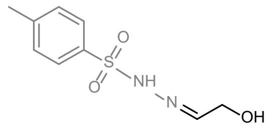
CH_2O	30.0100	199.0526	11.69	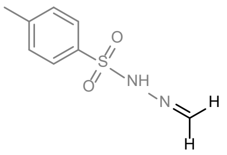

*Determined by IC chromatography.

Supplementary Table 2. The enthalpy of reaction of PhOH and representative oligomers.

	PhOH	$C_{12}H_{10}O_2$	$C_{18}H_{14}O_3$	$C_{24}H_{18}O_4$
Structure	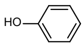	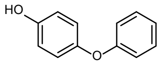	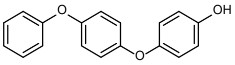	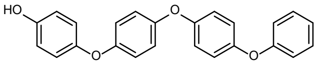
$\Delta_f H$ [a] (kJ mol^{-1})	-106.1	-169.8	-234.4	-298.7
Enthalpy of reaction [b] (MJ mol^{-1})	3.11	5.98	8.86	11.7

[a]. Enthalpy of formation. See Supplementary Method 3 for calculation details of enthalpy of formation.

[b]. The reaction of PhOH or oligomers can be represented by:

$C_{6x}H_{4x+2}O_x + (6.5x+0.5)O_2 \rightarrow 6xCO_2 + (2x+1)H_2O$

where x represents the ring number of oligomers. The enthalpy of reaction is calculated based on Hess's Law:

$$\Delta_f H \text{ (kJ mol}^{-1}) = \Sigma \Delta_f H \text{ products} - \Sigma \Delta_f H \text{ reactants}$$

$$= (2x+1)\times(-285.83) + 6x\times(-393.51) - \Delta_f H \text{ of } C_{6x}H_{4x+2}O_x$$

$$= -2932.72x - 285.83 - \Delta_f H \text{ of } C_{6x}H_{4x+2}O_x$$

Supplementary Table 3. The diffusion coefficients of the reactants and products.

		PhOH	$PhO^{\bullet}$	$C_{12}H_{10}O_2$	BQ
Collision diameter (nm)		0.57	0.57	0.8	0.57
Molecular diffusion coefficient ($m^2\ s^{-1}$)		7.87×10^{-10}	8.02×10^{-10}	5.29×10^{-10}	7.87×10^{-10}
K_n	5 nm	5.53	5.53	2.81	5.53
	20 nm	1.38	1.38	0.70	1.38
	55 nm	0.50	0.50	0.26	0.50
	120 nm	0.23	0.23	0.12	0.23
	1000 nm	0.03	0.03	0.01	0.03
Kunsden diffusion coefficient ($m^2\ s^{-1}$)	5 nm	4.28×10^{-10}	4.30×10^{-10}	3.04×10^{-10}	3.99×10^{-10}
	20 nm	1.72×10^{-9}	1.72×10^{-9}	1.72×10^{-9}	1.72×10^{-9}
	55 nm	4.71×10^{-9}	4.73×10^{-9}	3.35×10^{-9}	4.39×10^{-9}
	120 nm	1.03×10^{-8}	1.03×10^{-8}	7.30×10^{-9}	9.58×10^{-9}
	1000 nm	8.56×10^{-8}	8.60×10^{-8}	6.08×10^{-8}	7.99×10^{-8}
Effective diffusion coefficient ($m^2\ s^{-1}$)	5 nm	2.77×10^{-10}	2.8×10^{-10}	1.93×10^{-10}	2.65×10^{-10}
	20 nm	5.4×10^{-10}	5.47×10^{-10}	4.05×10^{-10}	5.4×10^{-10}
	55 nm	6.85×10^{-10}	6.86×10^{-10}	6.47×10^{-10}	6.67×10^{-10}
	120 nm	7.44×10^{-10}	7.44×10^{-10}	7.23×10^{-10}	7.27×10^{-10}
	1000 nm	7.95×10^{-10}	7.95×10^{-10}	7.92×10^{-10}	7.79×10^{-10}

Supplementary Table 4. Characteristics of the wastewater secondary effluents.

**Indexes**	**Textile dyeing secondary effluent**	**Chemical industry secondary effluent**
pH	7.80	7.58
TOC (mgC L^{-1})	15.2	39.8
Na^+ (mg L^{-1})	1147	1325
K^+ (mg L^{-1})	26.0	31.2
Ca^{2+} (mg L^{-1})	42.6	62.4
Mg^{2+} (mg L^{-1})	3.6	63.3
SO_4^{2-} (mg L^{-1})	1331	1738
Cl^- (mg L^{-1})	1429	2755
NO_3^- (mg L^{-1})	154	40.4

Supplementary Table 5. UHPLC operational conditions for different organic compounds.

Substrates	Flow (mL/min)	λ (nm)	Methanol (%)	0.1% HCOOH Solution (%)	Acetonitrile (%)	Column
PhOH	0.2	270	50	50	—	C18, 50×2.1 mm, 1.9 μm particle size
PMSO	0.2	215	—	80	20	
$PMSO_2$	0.2	215	—	80	20	
4-chlorophenol	0.2	280	50	50	—	
aniline	0.2	260	30	70	—	
bisphenol A	0.2	230	65	35	—	

Supplementary Table 6. Operational conditions of UHPLC-MS/MS analysis.

Parameter	ESI$^-$	ESI$^+$
Column temperature	25 °C	
Sampler temperature	9 °C	
Injection volume	20 μL	
Flow rate	0.2 mL min^{-1}	
λ	230 nm	—
Mobile phase	A: H_2O<br>B: Methanol	A: 0.1% formic acid in H_2O<br>B: Methanol
Elution program	0–2 min, 2% B<br>2–12 min, 2%–50% B<br>12–18 min, 50% B<br>18–18.5 min, 50%–2% B<br>18.5–21 min, 2% B	0–2 min, 2% B<br>2-10 min, 2%–60% B<br>10-17 min, 60% B<br>17-17.5 min, 60%–2% B<br>17.5-20 min, 2% B
Scan mode	Full mass-DDA	
Resolution	MS^1: 70000, MS^2: 17500	
AGC target	MS^1: 3×10^6, MS^2: 1×10^5	
Normalized collision energy	10, 30, 60 eV	
Sheath gas pressure	35 arb	
Aux gas pressure	10 arb	
Sweep gas pressure	1 arb	
HESI spray voltage	+3.5 kV	-3.0 kV
Capillary temperature	350 °C	
Aux gas heated temperature	400 °C	
S-lens RF level	50	
Isolation window	1.5 m/z	
Dynamic exclusion	6.0 s	

Supplementary Figures

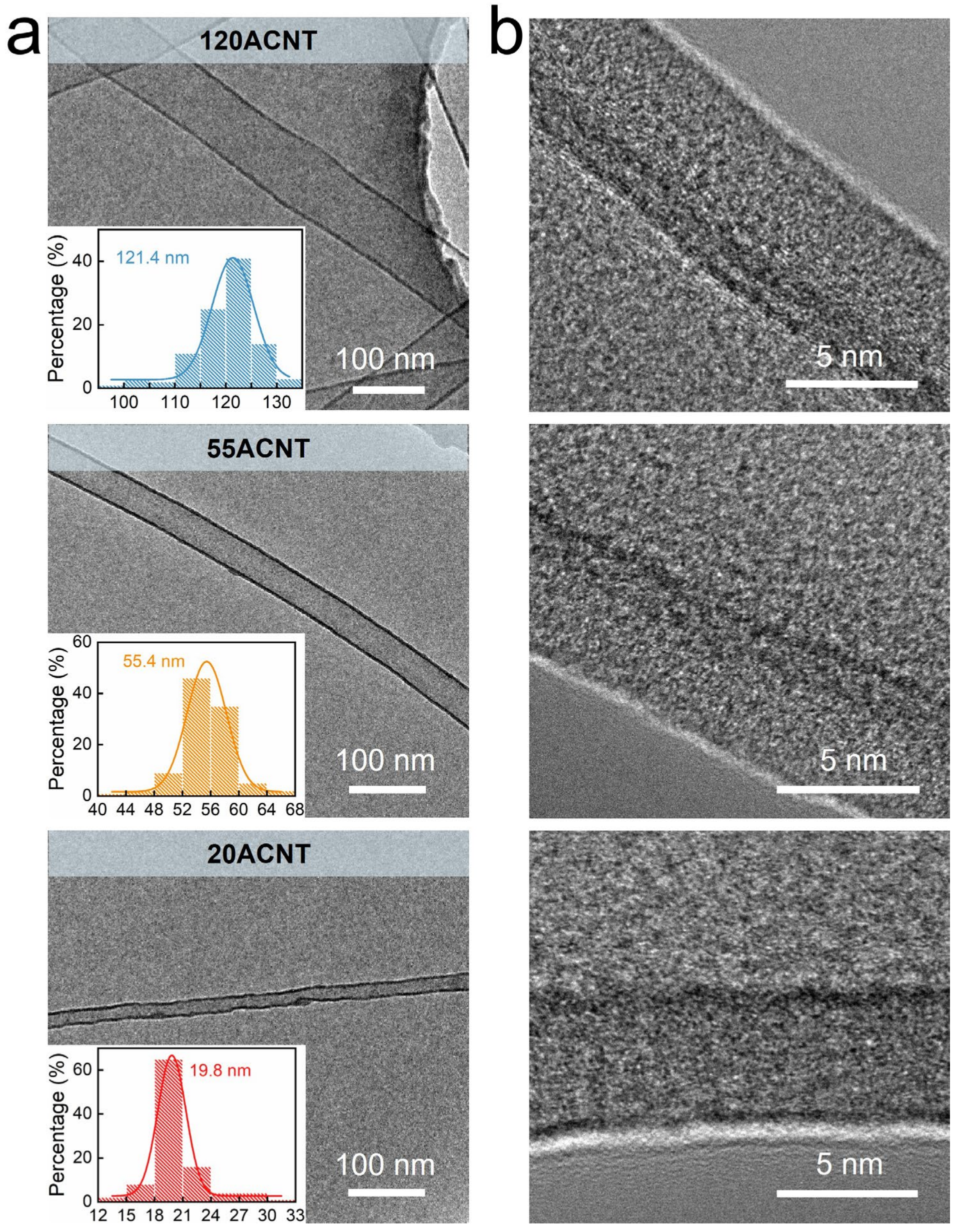

Supplementary Fig. 1 TEM images of ACNTs at different scale bars. The insets in Fig. 1a show the distribution of pore sizes of different samples.

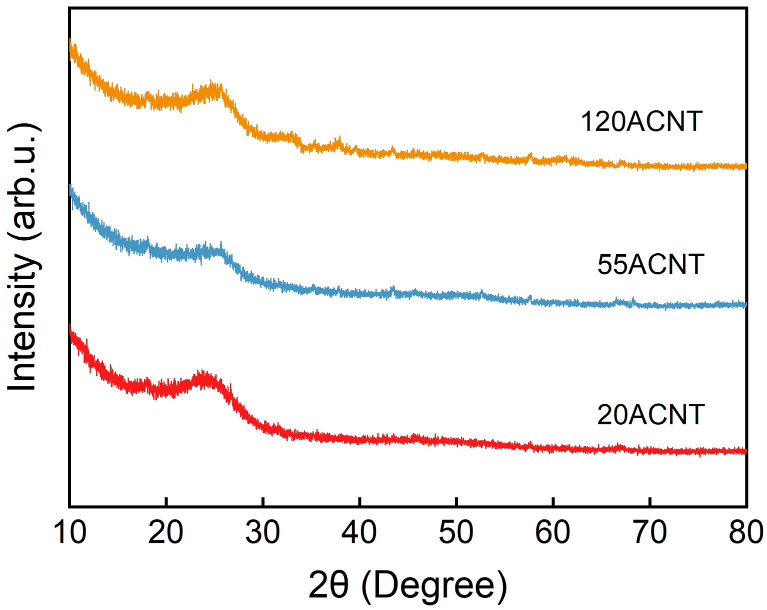

Supplementary Fig. 2 XRD spectra of three ACNTs.

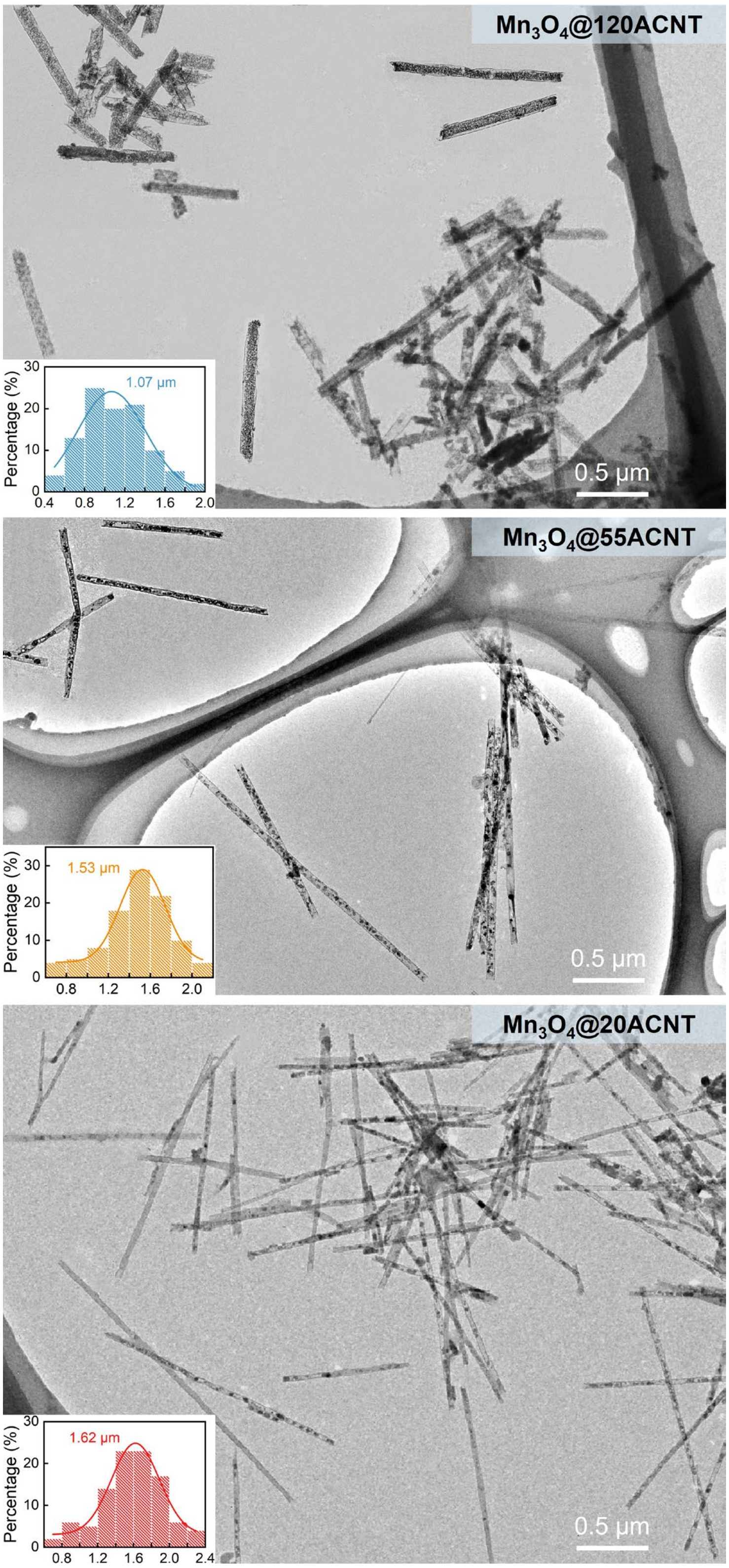

Supplementary Fig. 3 TEM images of Mn3O4@nACNT. The insets show the histogram of the length distribution of the catalysts.

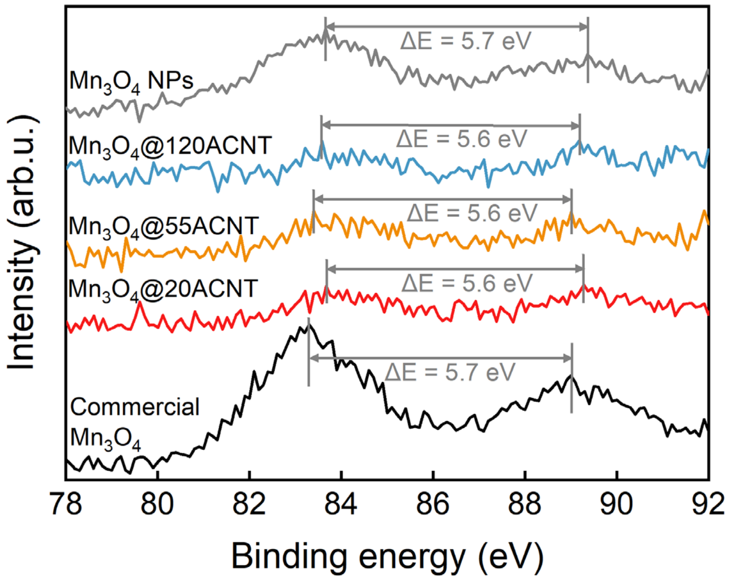

Supplementary Fig. 4 Mn 3*s* XPS spectra of Mn_3O_4@*n*ACNT and bulk Mn_3O_4 NPs.

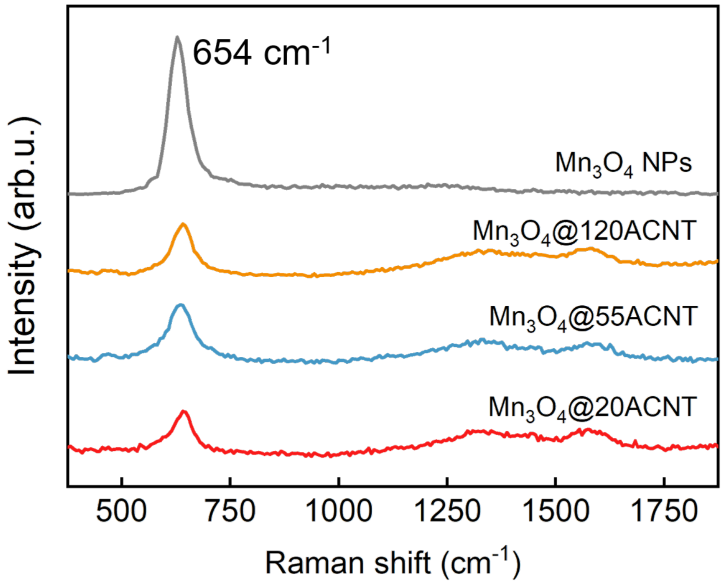

Supplementary Fig. 5 Raman spectra of Mn_3O_4@*n*ACNT and bulk Mn_3O_4 NPs.

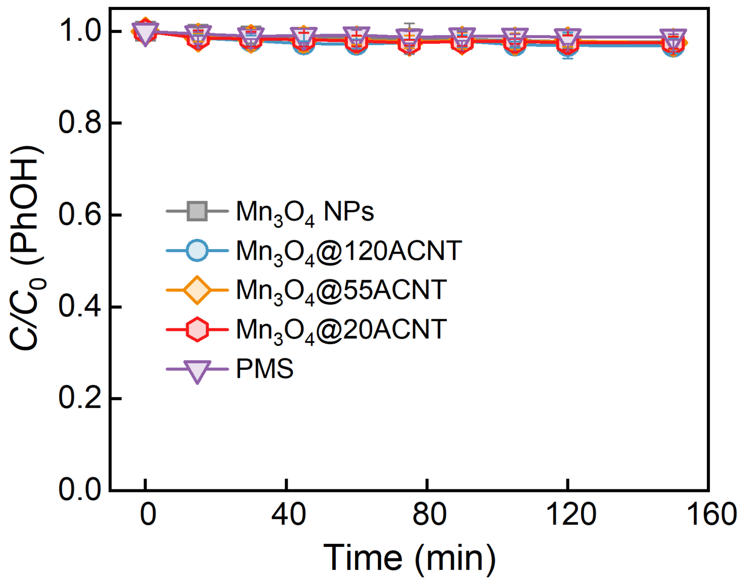

Supplementary Fig. 6 Conversion of PhOH in the absence of either PMS or catalysts. Conditions: T = 293.2±0.3 K; pH = 7.0±0.1; [PhOH] = 200 μM; [catalyst] = 0/75 mg L^{-1}; [PMS] = 0/2.0 mM. The error bars represent the standard deviations from triplicate tests.

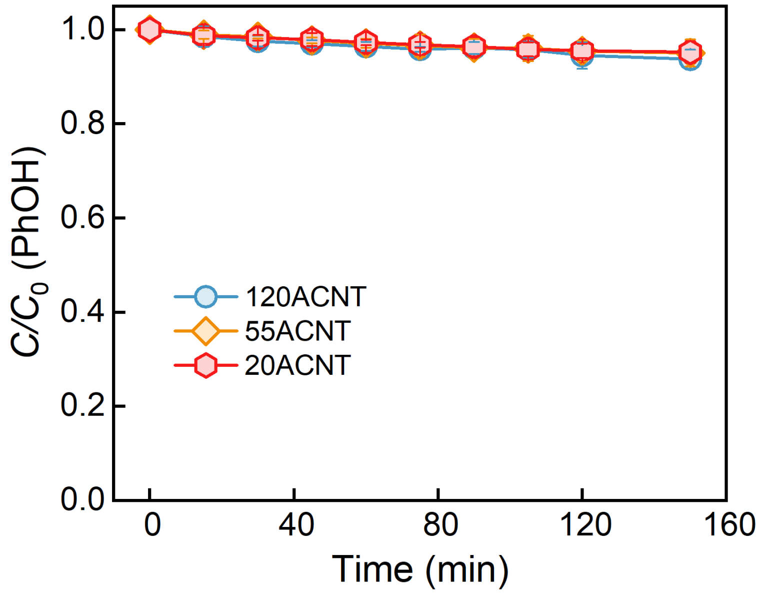

Supplementary Fig. 7 Conversion of PhOH in *n*ACNT/PMS systems. Conditions: T = 293.2±0.3 K; pH = 7.0±0.1; [PhOH] = 200 μM; [catalyst] = 75 mg L^{-1}; [PMS] = 2.0 mM. The error bars represent the standard deviations from triplicate tests.

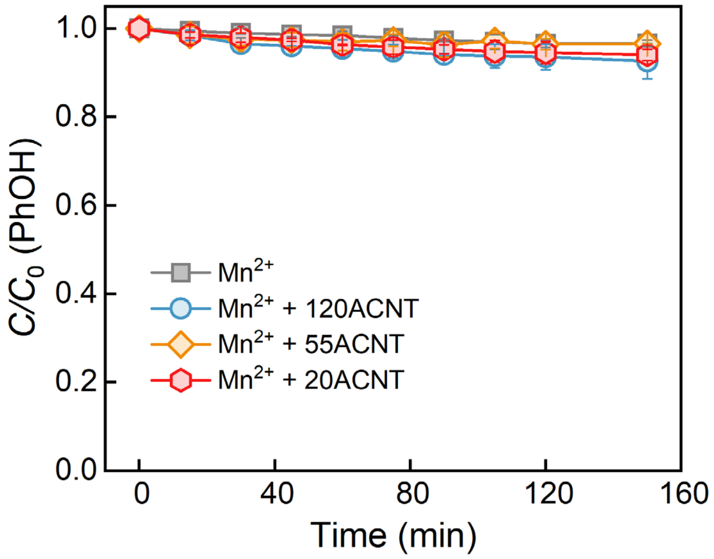

Supplementary Fig. 8 Effect of Mn^{2+} leaching on the conversion of PhOH. Conditions: T = 293.2±0.3 K; pH = 7.0±0.1; [PhOH] = 200 μM; [Mn^{2+}] = 20 μM; [catalyst] = 75 mg L^{-1}; [PMS] = 2.0 mM. The error bars represent the standard deviations from triplicate tests.

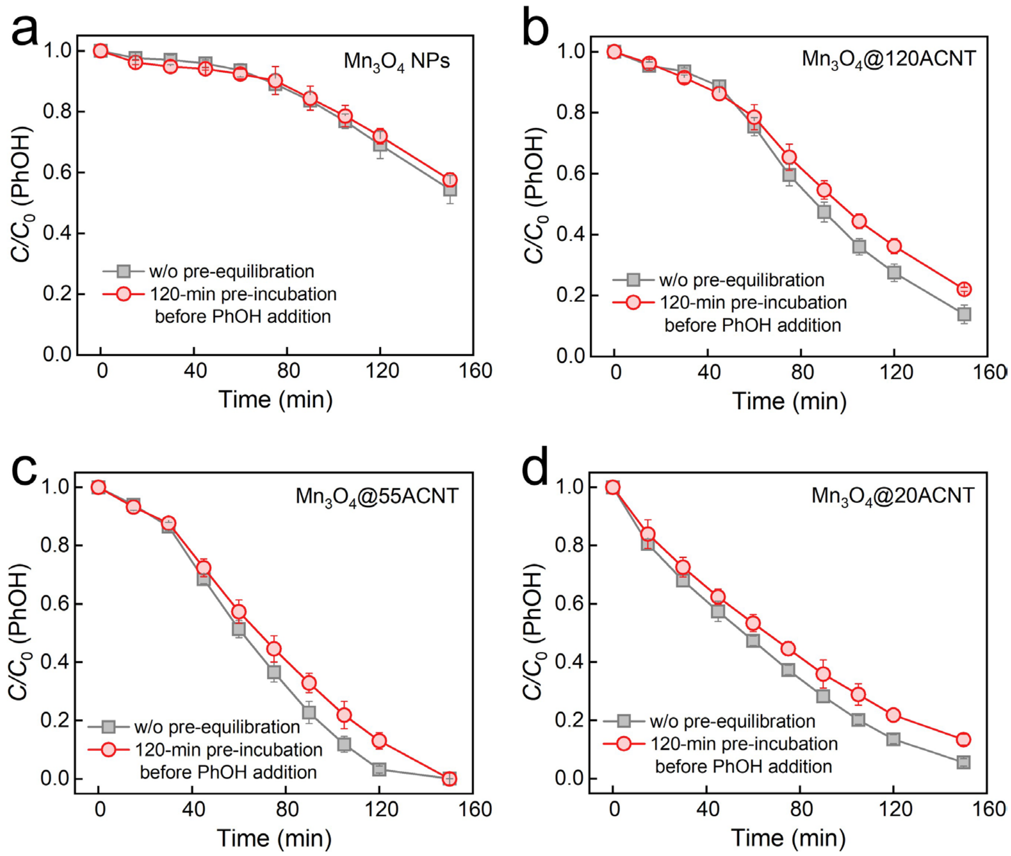

Supplementary Fig. 9 Effect of pre-equilibration of the catalysts and PMS on PhOH conversion in different oxidation systems. Conditions: T = 293.2±0.3 K; pH = 7.0±0.1; [PhOH] = 200 μM; [catalyst] = 75 mg L^{-1}; [PMS] = 2.0 mM. The error bars represent the standard deviations from triplicate tests.

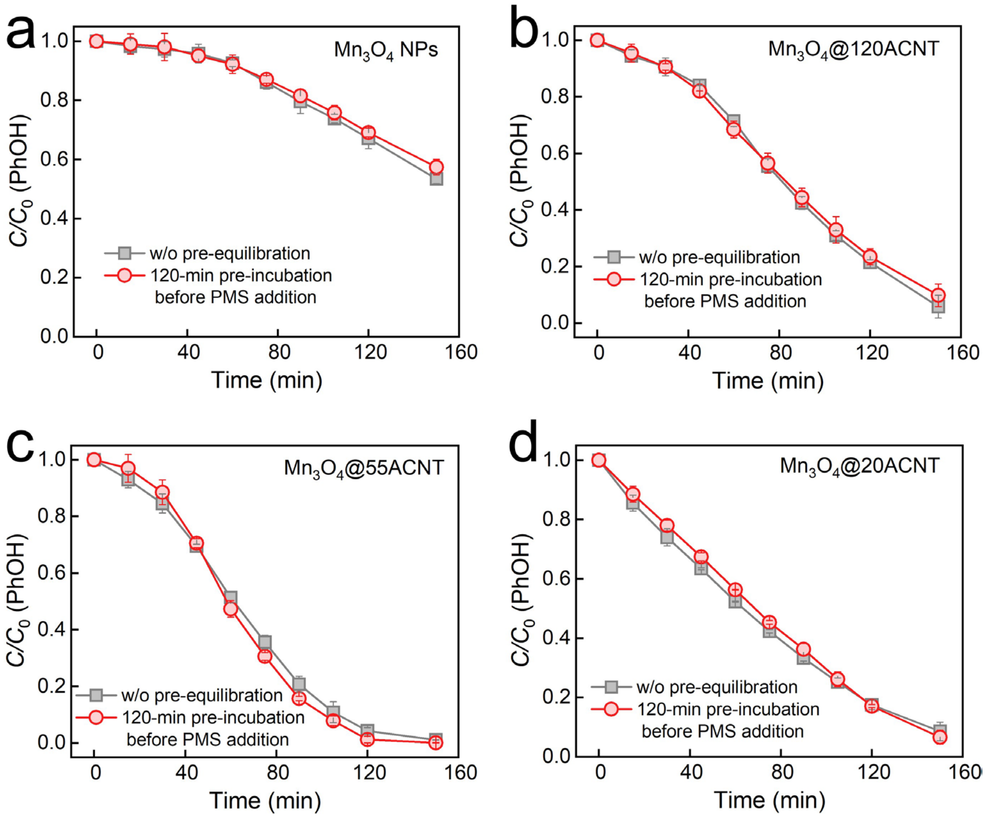

Supplementary Fig. 10 Effect of pre-equilibration of the catalysts and PhOH on PhOH conversion in different oxidation systems. Conditions: T = 293.2±0.3 K; pH = 7.0±0.1; [PhOH] = 200 μM; [catalyst] = 75 mg L^{-1}; [PMS] = 2.0 mM. The error bars represent the standard deviations from triplicate tests.

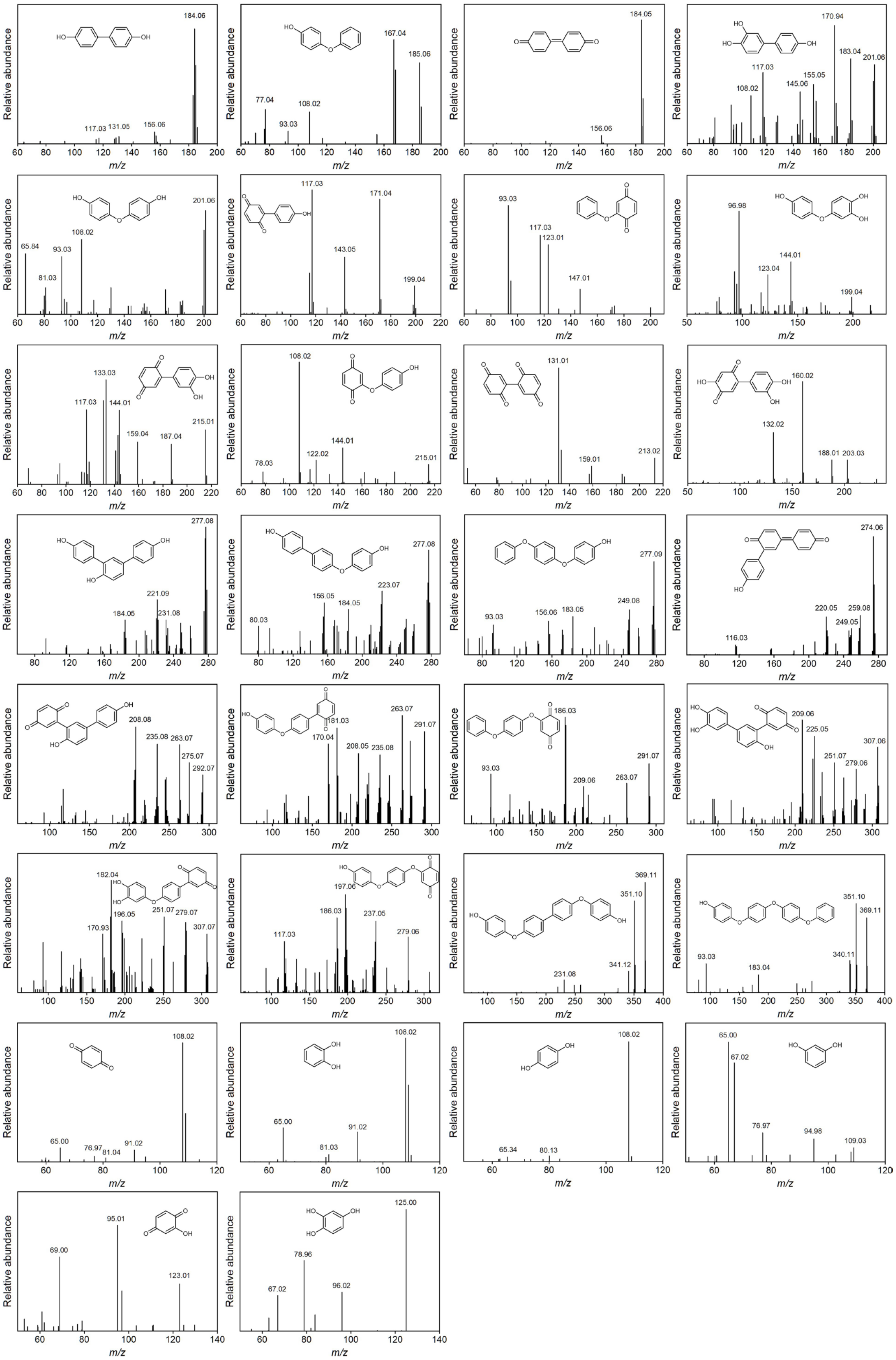

Supplementary Fig. 11 The UHPLC-MS/MS spectra of oligomers and hydroxylated products detected in the oxidation processes.

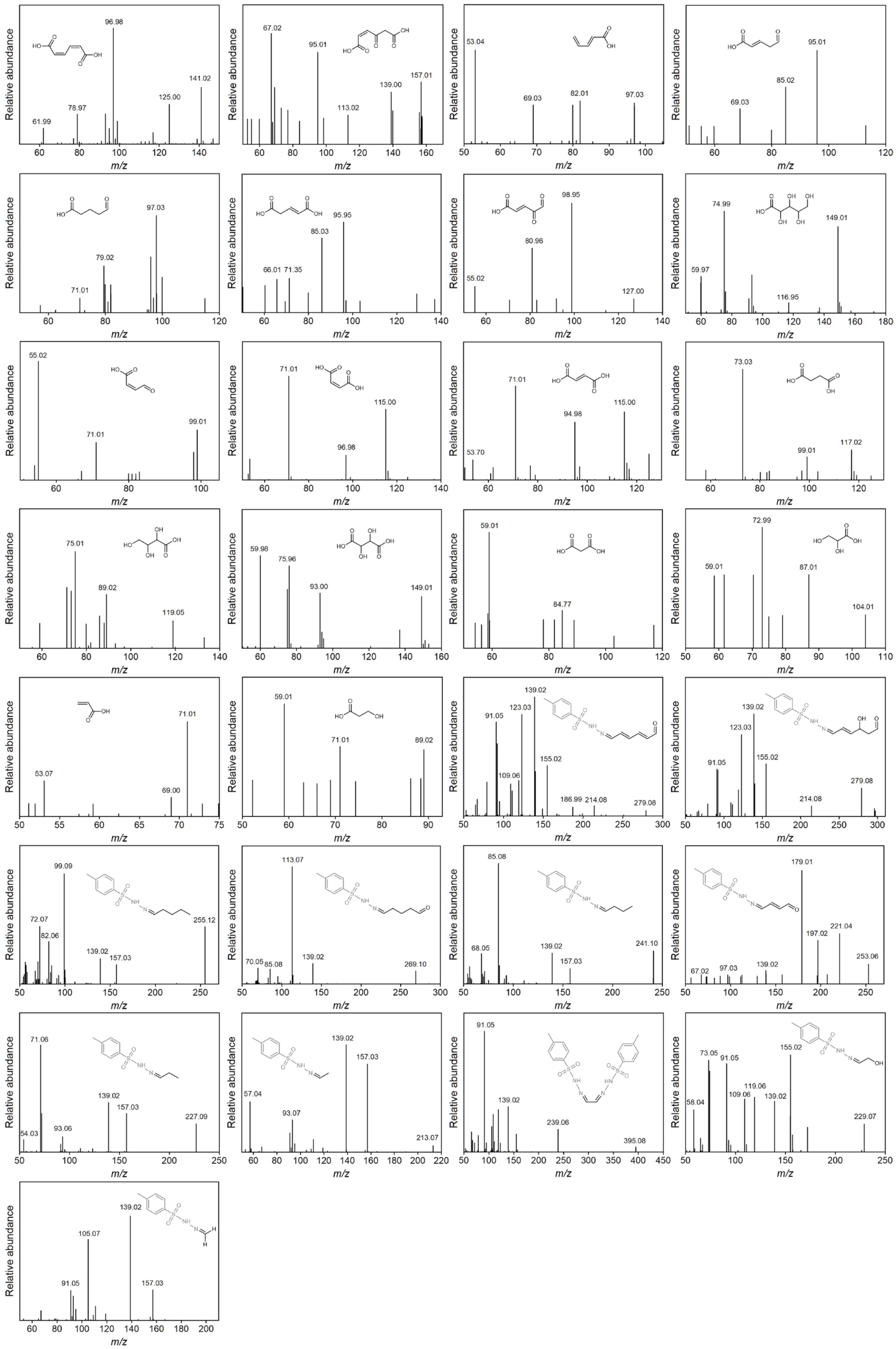

Supplementary Fig. 12 The UHPLC-MS/MS spectra of organic acids and carbonyl compounds detected in the oxidation processes.

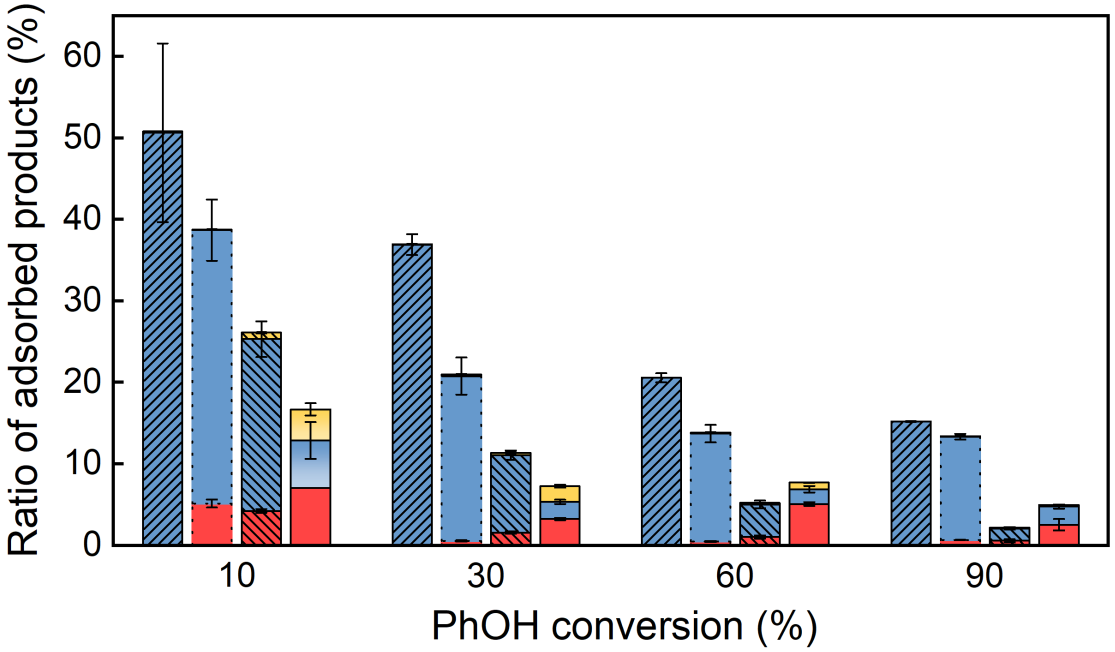

Supplementary Fig. 13 The ratio of products adsorbed on the catalysts to the total products generated. The error bars represent the standard deviations from triplicate tests.

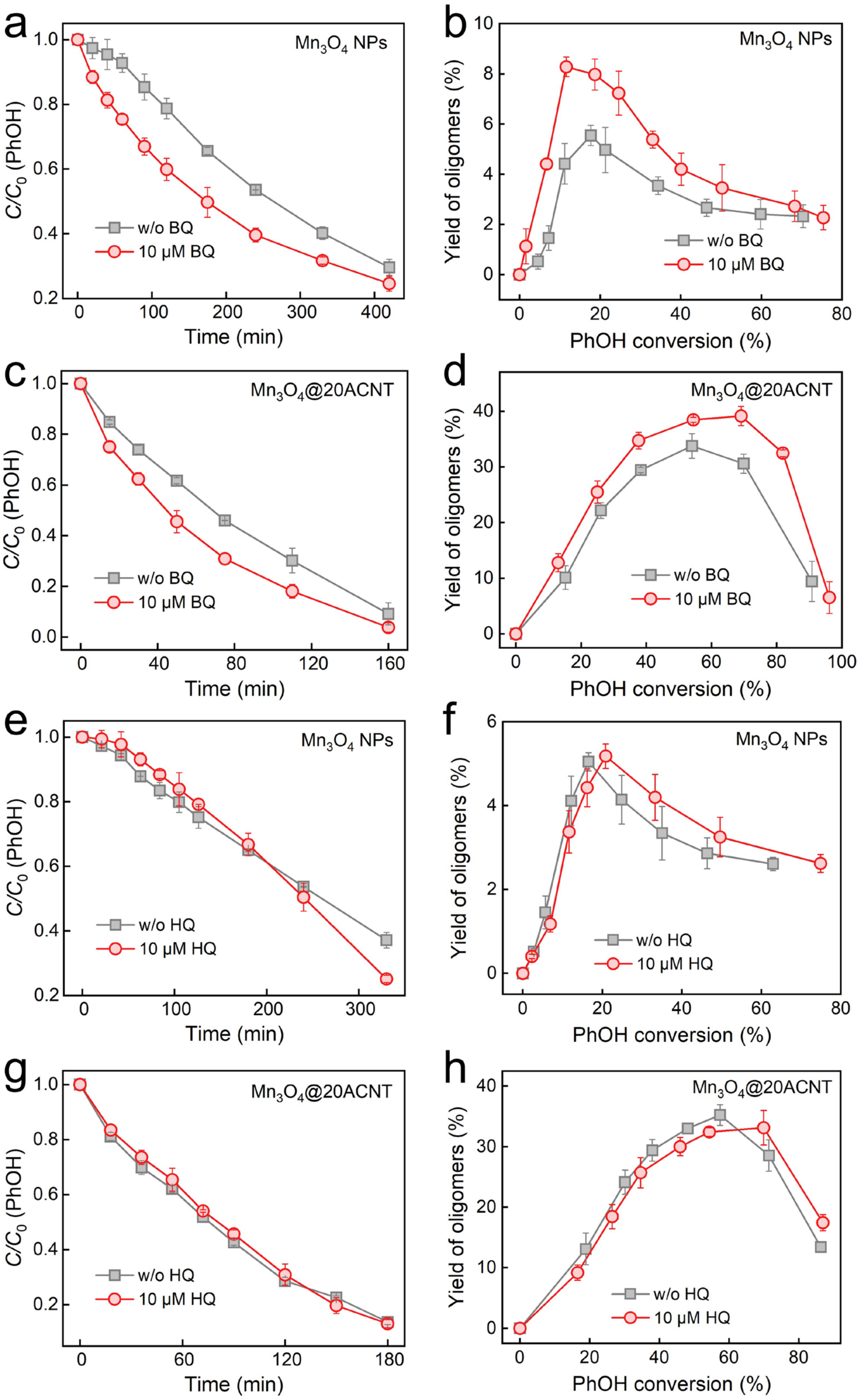

Supplementary Fig. 14 Effect of BQ and HQ on PhOH conversion and oligomer yield in bulk Mn_3O_4/PMS and Mn_3O_4@20ACNT/PMS. Conditions: T = 293.2±0.3 K; pH = 7.0±0.1; [PhOH] = 200 μM; [BQ]= [HQ] = 10 μM; [catalyst] = 75 mg L^{-1}; [PMS] = 2.0 mM. The error bars represent the standard deviations from triplicate tests.

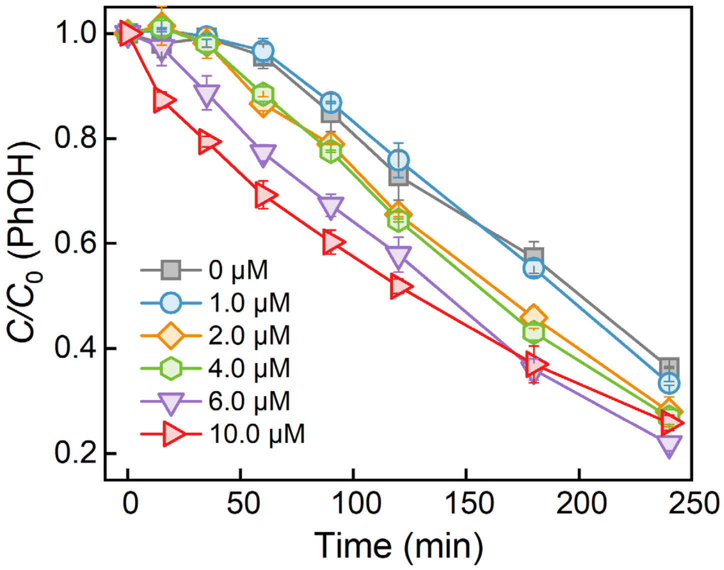

Supplementary Fig. 15 Effect of BQ concentration on PhOH conversion in bulk Mn_3O_4/PMS. Conditions: T = 293.2±0.3 K; pH = 7.0±0.1; [PhOH] = 200 μM; [PMS] = 2.0 mM; [catalyst] = 75 mg L^{-1}. The error bars represent the standard deviations from triplicate tests.

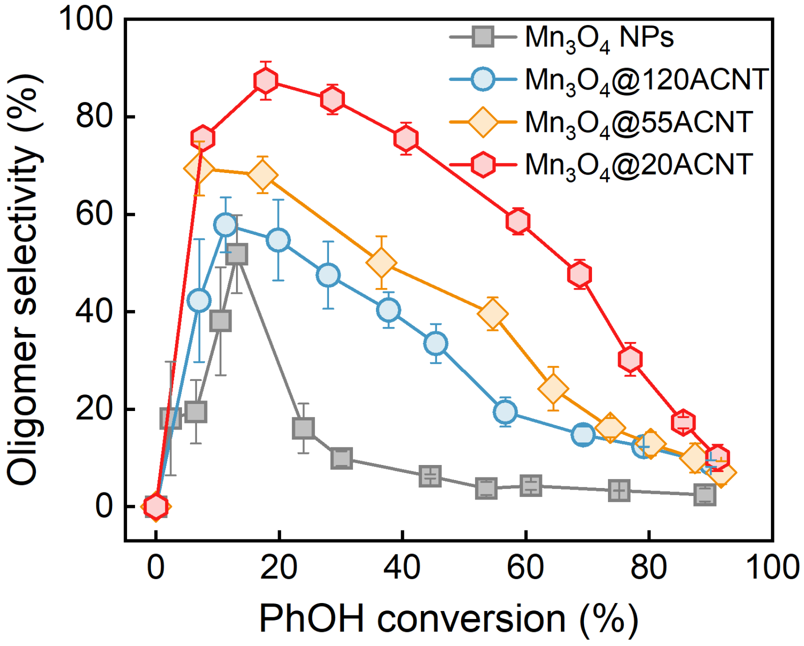

Supplementary Fig. 16 Reaction selectivity towards oligomers at different PhOH conversions in four oxidation systems. Conditions: T = 293.2±0.3 K; pH = 7.0±0.1; [PhOH] = 200 μM; [PMS] = 2.0 mM; [catalyst] = 75 mg L^{-1}. The error bars represent the standard deviations from triplicate tests.

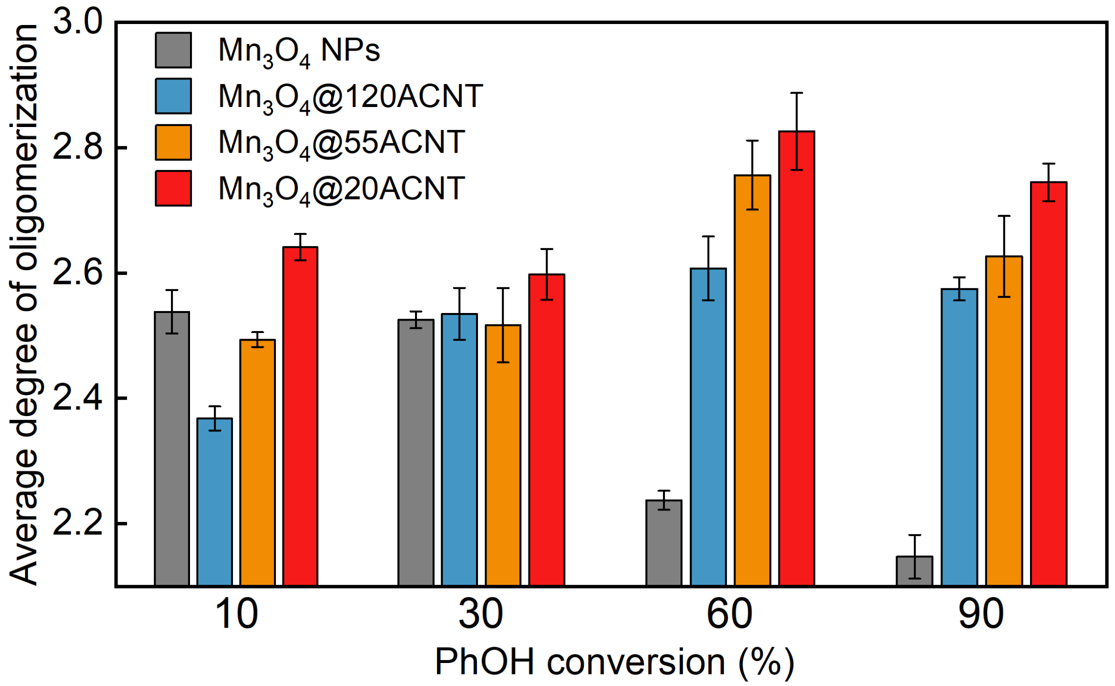

Supplementary Fig. 17 The average degree of oligomerization at different PhOH conversions in four oxidation systems. Conditions: T = 293.2±0.3 K; pH = 7.0±0.1; [PhOH] = 200 μM; [PMS] = 2.0 mM; [catalyst] = 75 mg L^{-1}. The error bars represent the standard deviations from triplicate tests.

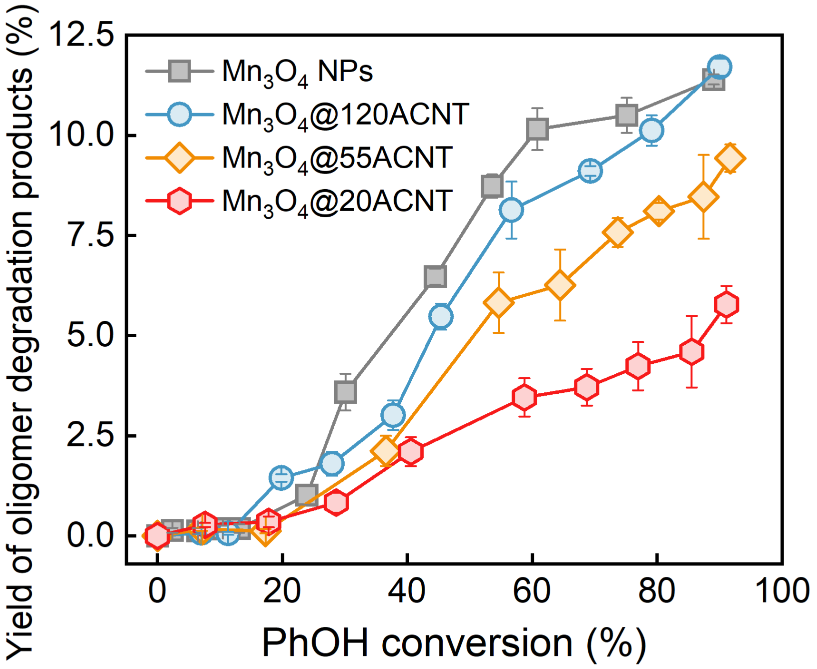

Supplementary Fig. 18 Yields of the $C_{7\text{-}11}$ degradation products at different PhOH conversions in four oxidation systems. Conditions: T = 293.2±0.3 K; pH = 7.0±0.1; [PhOH] = 200 μM; [PMS] = 2.0 mM; [catalyst] = 75 mg L^{-1}. The error bars represent the standard deviations from triplicate tests.

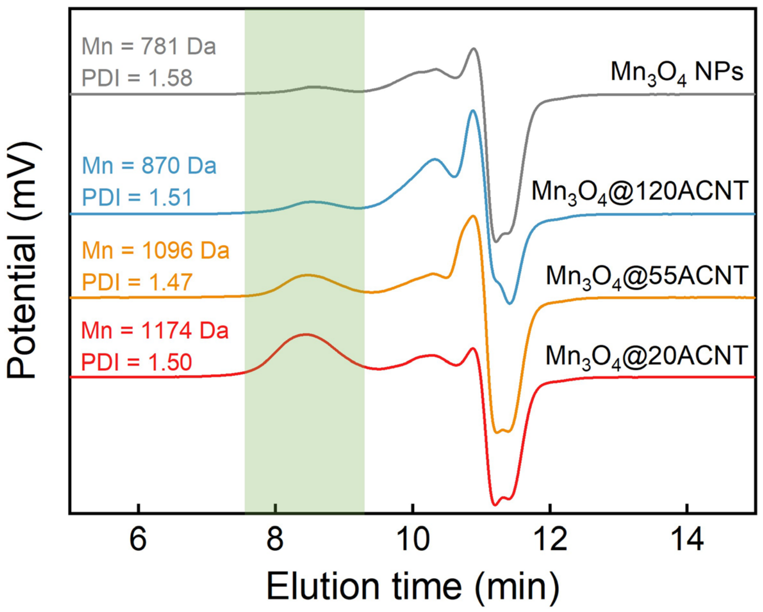

Supplementary Fig. 19 GPC spectra of the products extracted from the reacted catalysts. The green shadow indicates the region of eluted polymers.

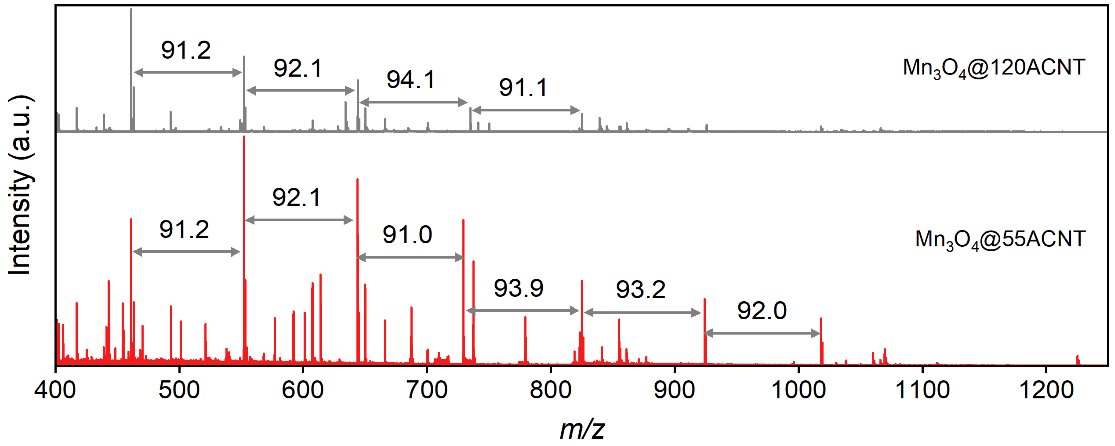

Supplementary Fig. 20 MALDI-TOF-MS spectra of products adsorbed on the catalysts in Mn_3O_4@55ACNT/PMS and Mn_3O_4@120ACNT/PMS.

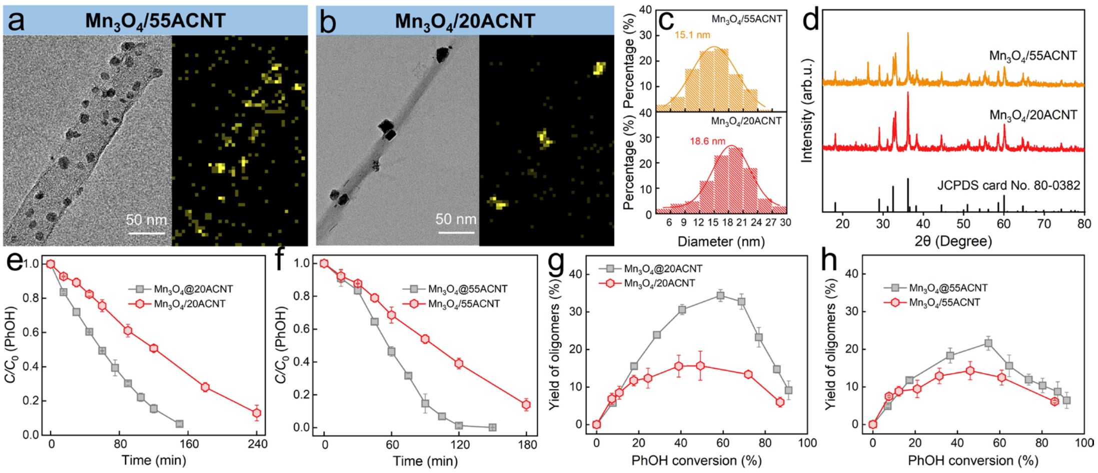

Supplementary Fig. 21 Characterization of Mn_3O_4/*n*ACNT and PhOH conversion in Mn_3O_4/nACNT/PMS. TEM images and EDX elemental mappings of (a) Mn_3O_4/55ACNT and (b) Mn_3O_4/20ACNT. (c) Size-distribution histograms of Mn_3O_4 NPs in different catalysts. (d) XRD patterns of the catalysts. (e–f) The kinetics of PhOH degradation in Mn_3O_4/*n*ACNT/PMS. (g–h) The yield of oligomers in Mn_3O_4/*n*ACNT/PMS. The Mn contents of Mn_3O_4/20ACNT and Mn_3O_4/55ACNT were 15.5 wt% and 24.4 wt%, respectively. Conditions: T = 293.2±0.3 K; pH = 7.0±0.1; [PhOH] = 200 μM; [PMS] = 2.0 mM; [catalyst] = 75 mg L^{-1}. The error bars represent the standard deviations from triplicate tests.

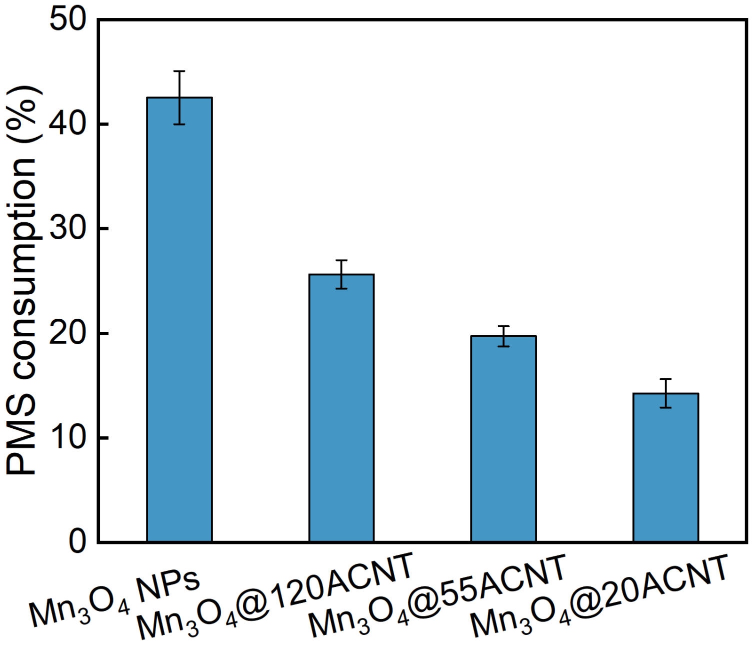

Supplementary Fig. 22 Consumption of PMS in different oxidation systems. Conditions: T = 293.2±0.3 K; [PhOH] = 200 μM; [PMS] = 2.0 mM; [catalyst] = 75 mg L^{-1}. The error bars represent the standard deviations from triplicate tests.

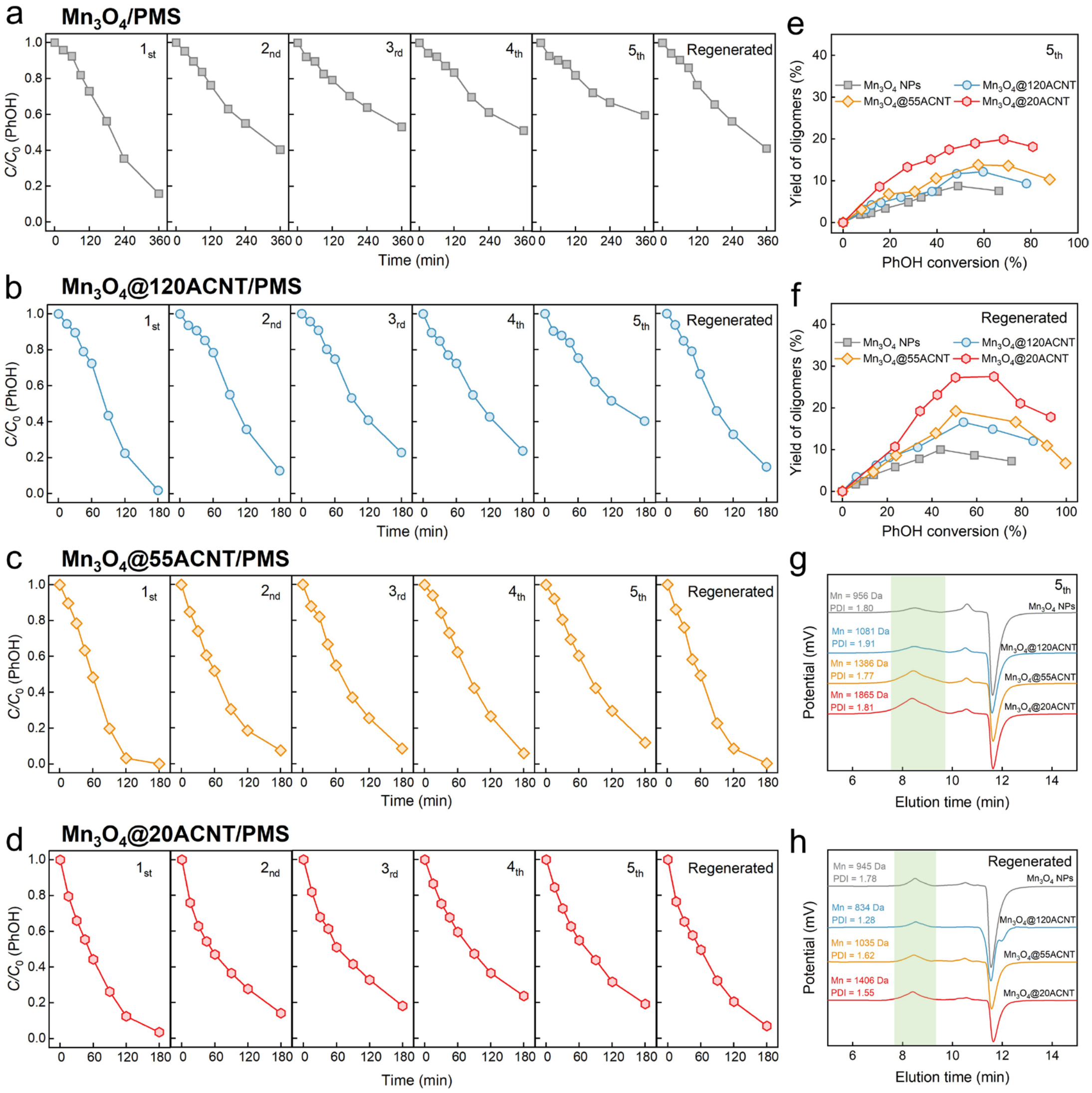

Supplementary Fig. 23 The reusability of the catalysts. The kinetics of PhOH degradation during five continuous runs and after regeneration in (a) Mn_3O_4/PMS and (b–d) Mn_3O_4@*n*ACNT/PMS. Plots of the yield of oligomers versus PhOH conversion after (e) five continuous runs and (f) regeneration in different oxidation systems. (i) GPC spectra of the products extracted from Mn_3O_4 NPs and Mn_3O_4@*n*ACNT after (g) five continuous runs and (h) regeneration. Conditions: T = 293.2±0.3 K; pH = 7.0±0.1; [PhOH] = 200 μM; [PMS] = 2.0 mM; [catalyst] = 75 mg L^{-1}. The green shadow in Fig. 23g–h indicates the region of eluted polymers.

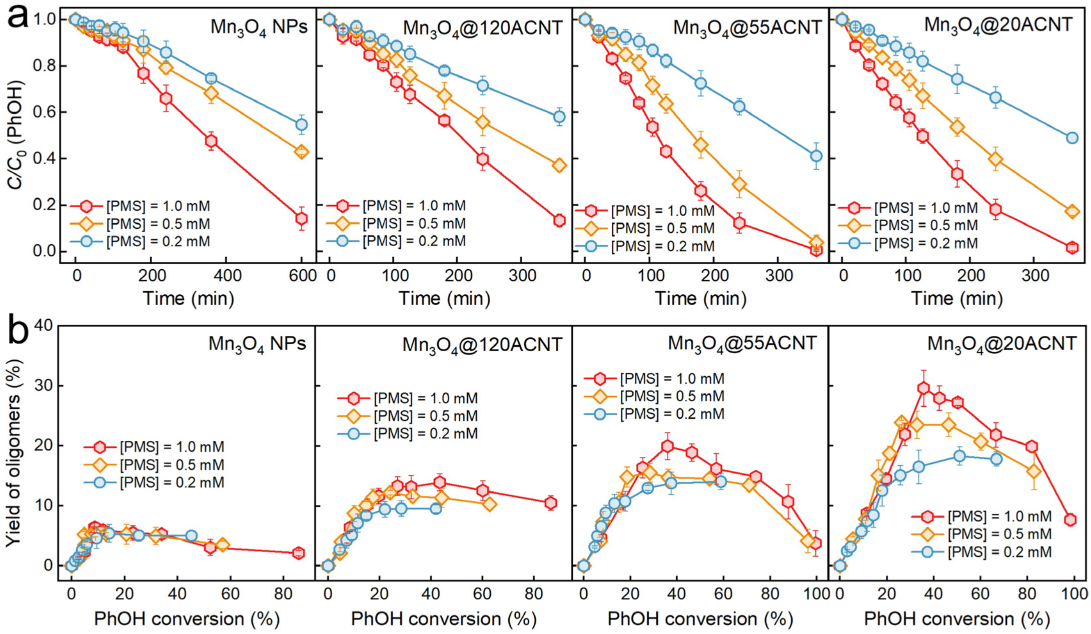

Supplementary Fig. 24 Effect of PMS concentration on (a) the kinetics of PhOH degradation and (b) the oligomer yield in different oxidation systems. Conditions: T = 293.2±0.3 K; pH = 7.0±0.1; [PhOH] = 200 μM; [catalyst] = 75 mg L^{-1}. The error bars represent the standard deviations from triplicate tests.

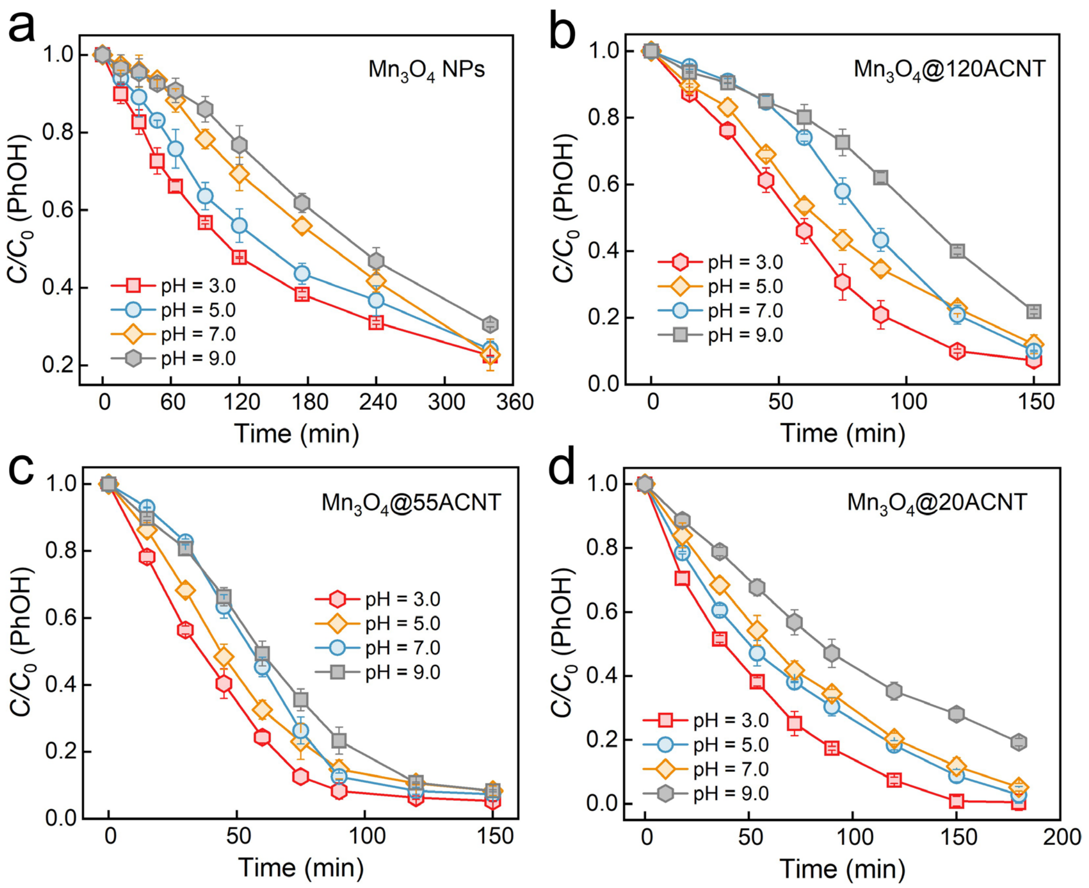

Supplementary Fig. 25 Effect of pH on PhOH conversion in (a) Mn_3O_4/PMS, (b) Mn_3O_4@120ACNT/PMS, (c) Mn_3O_4@55ACNT/PMS, and (d) Mn_3O_4@20ACNT/PMS. Conditions: T = 293.2±0.3 K; [PhOH] = 200 μM; [PMS] = 2.0 mM; [catalyst] = 75 mg L^{-1}. The error bars represent the standard deviations from triplicate tests.

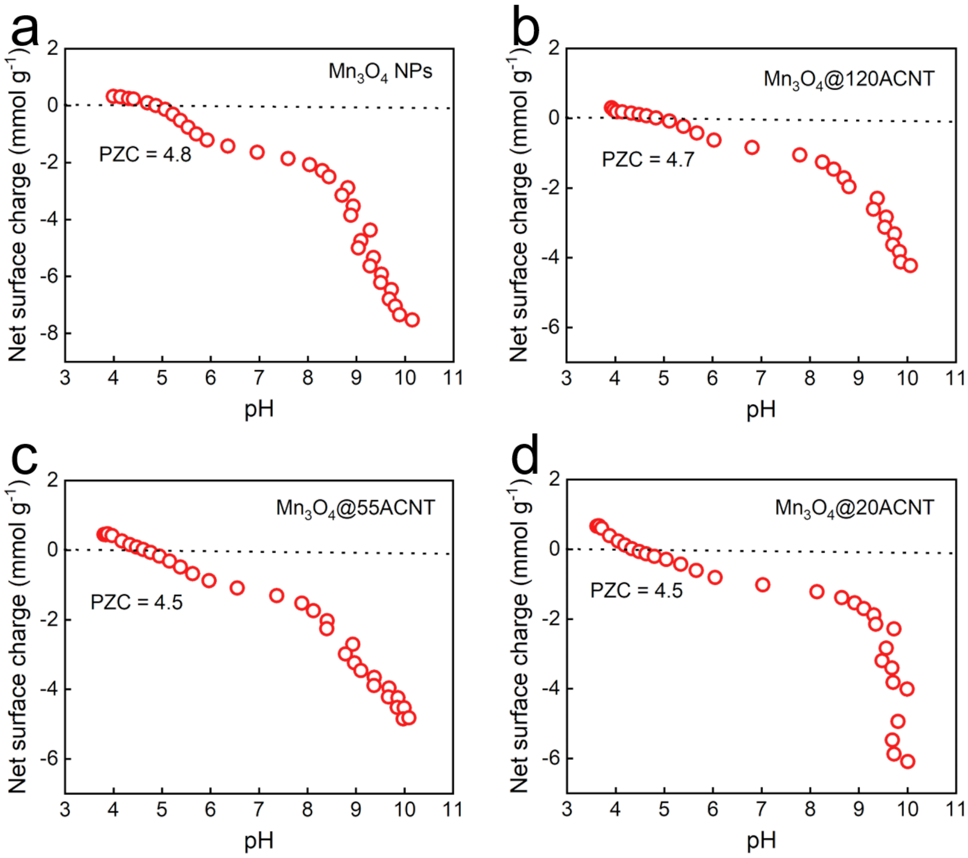

Supplementary Fig. 26 Plots of the net surface charge of (a) Mn_3O_4 NPs, (b) Mn_3O_4@120ACNT, (c) Mn_3O_4@55ACNT, and (d) Mn_3O_4@20ACNT versus the solution pH values. The net surface charge (Qh) is the total concentration of protons captured by the surface. $Q_h = \frac{V_{acid}C_{acid} - V_{base}C_{base} - [H^+]V_{total} + [OH^-]V_{total}}{m}$, where V_{acid} is the total volume of acid to adjust the solution pH to lower side (pH 3.5–3.8); C_{acid} is the concentration of acid; V_{base} is the volume of base used to increase the solution pH; C_{base} is the concentration of base; V_{total} is the total volume of solution, and m represents the mass of the catalyst.

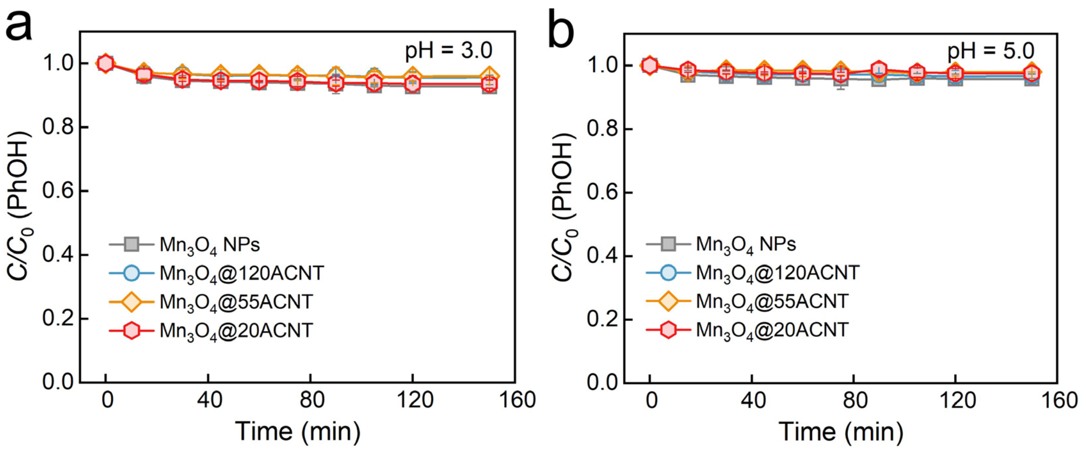

Supplementary Fig. 27 Removal of PhOH by different catalysts in the absence of PMS at pH 3.0 and 5.0. Conditions: T = 293.2±0.3 K; [PhOH] = 200 μM; [catalyst] = 75 mg L^{-1}. The error bars represent the standard deviations from triplicate tests.

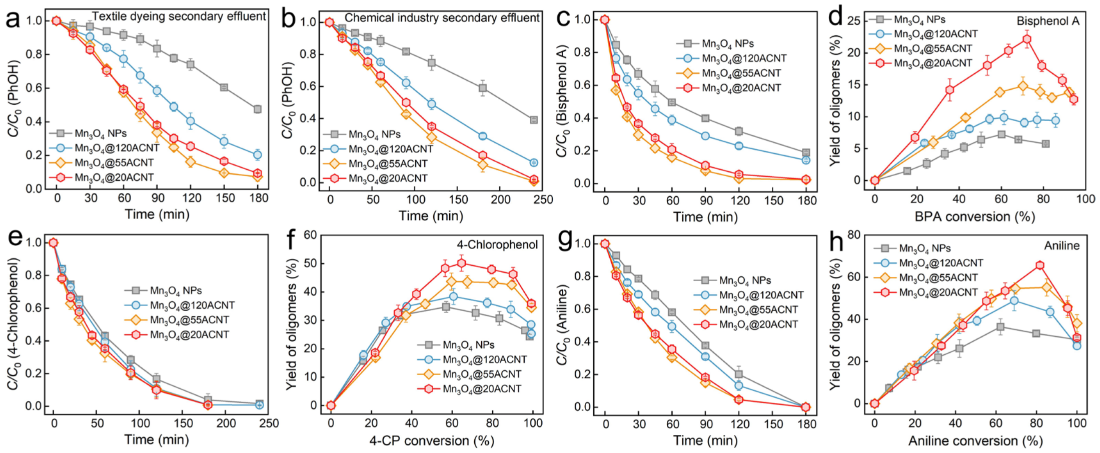

Supplementary Fig. 28 Kinetics of PhOH conversion in the secondary effluent of (a) textile dyeing and (b) chemical industry wastewater treatment processes. (c–h) The oxidation kinetics of bisphenol A, 4-chlorophenol, and aniline, and the corresponding yield of oligomers in different oxidation systems. Conditions: T = 293.2±0.3 K; pH = 7.0±0.1; [PhOH] = [aniline] = [4-chlorophenol] = [bisphenol A] = 200 μM; [PMS] = 2.0 mM; [catalyst] = 75 mg L^{-1}. The error bars represent the standard deviations from triplicate tests.

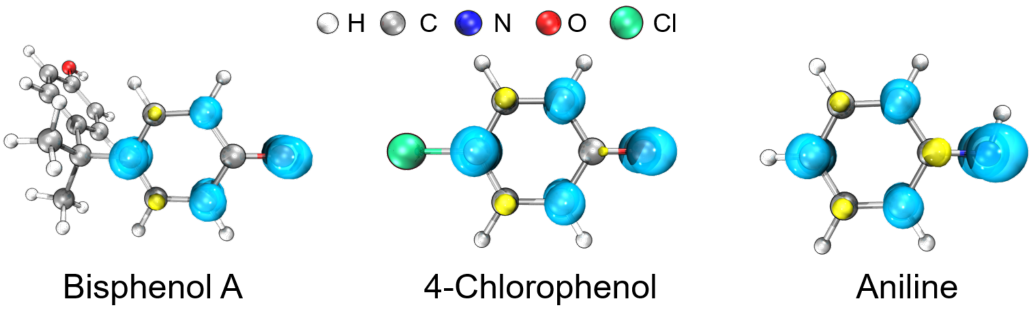

Supplementary Fig. 29 Isosurface map of the electron spin density of the radicals generated from oxidation of bisphenol A, 4-chlorophenol, and aniline. The blue and yellow shadows indicate the positive and negative spin density, respectively. See Supplementary Method 3 for detailed calculation. The unpaired electron is most likely located on the hydroxyl oxygen of bisphenol A and 4-chlorophenol, the amino nitrogen of aniline, and the *ortho* and *para* carbon atoms of the benzene ring. However, some of the sites, such as the *para* carbon of bisphenol A, seem unsuitable for the coupling reactions due to the steric effect.

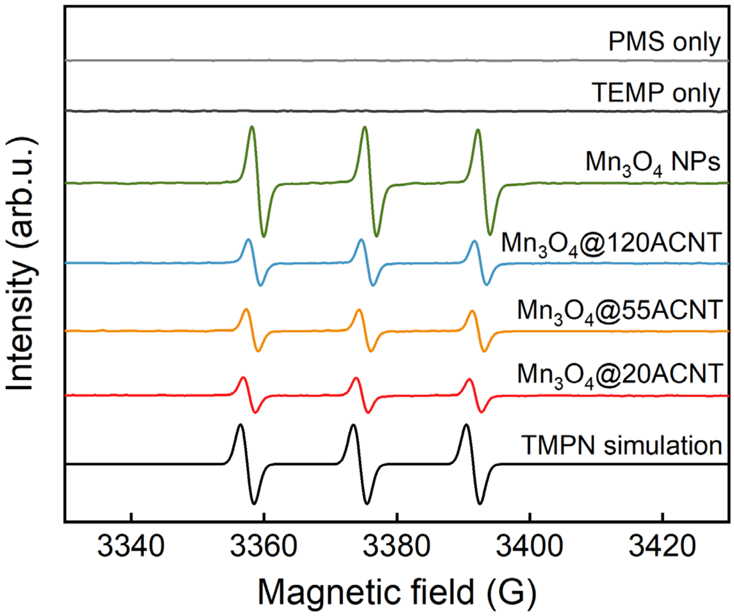

Supplementary Fig. 30 TEMP-trapped EPR spectra of different oxidation systems. Conditions: T = 293.2±0.3 K; pH = 7.0±0.1; [TEMP] = 100 mM; [PhOH] = 200 μM; [PMS] = 2.0 mM; [catalyst] = 75 mg L^{-1}.

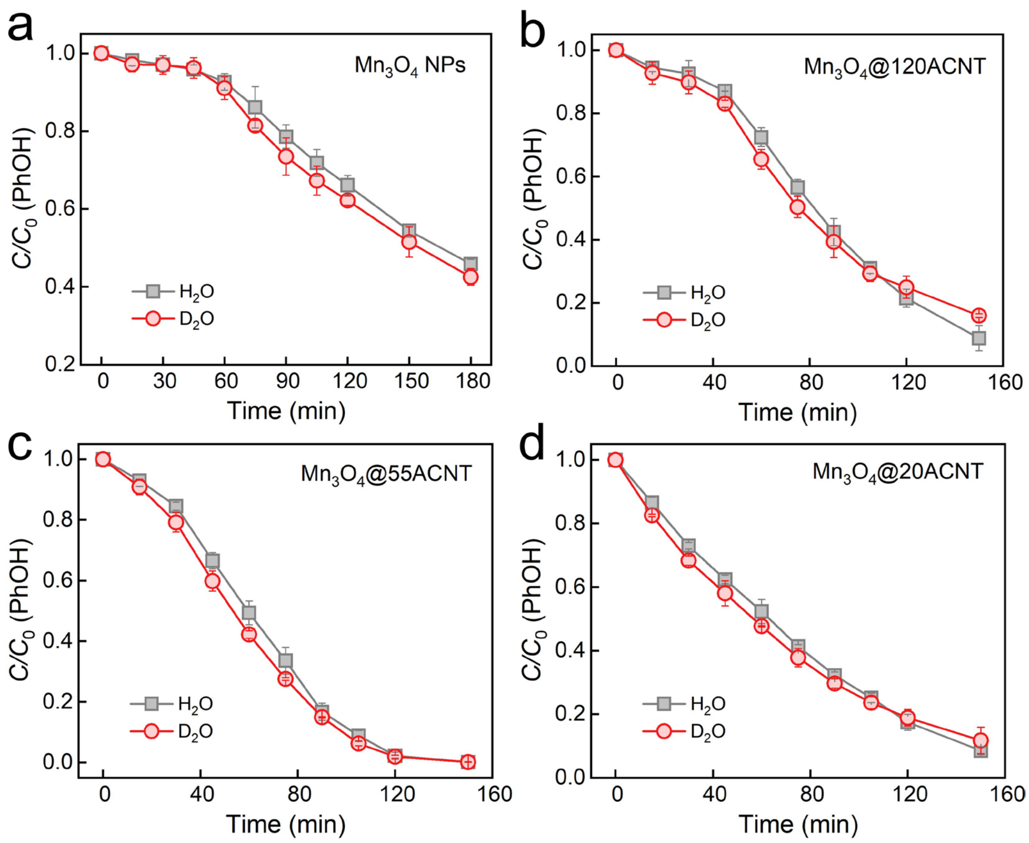

Supplementary Fig. 31 Effect of solvents on the conversion of PhOH in different oxidation systems. Conditions: T = 293.2±0.3 K; pH = 7.0±0.1; pD = 7.4±0.1; [PhOH] = 200 μM; [PMS] = 2.0 mM; [catalyst] = 75 mg L^{-1}. The error bars represent the standard deviations from triplicate tests.

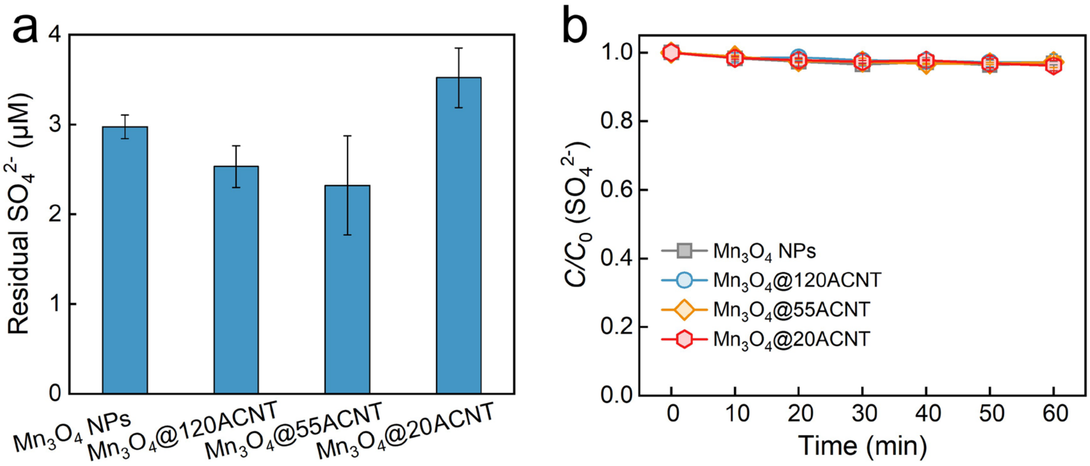

Supplementary Fig. 32 (a) The concentration of dissolved SO_4^{2-} after oxidation of PhOH by pre-oxidized catalysts. (b) Adsorption of SO_4^{2-} by different catalysts. Conditions: T = 293.2±0.3 K; pH = 7.0±0.1; [PhOH] = 200 μM; [catalyst] = 400 mg L^{-1}; [SO_4^{2-}] = 10 μM. The error bars represent the standard deviations from triplicate tests.

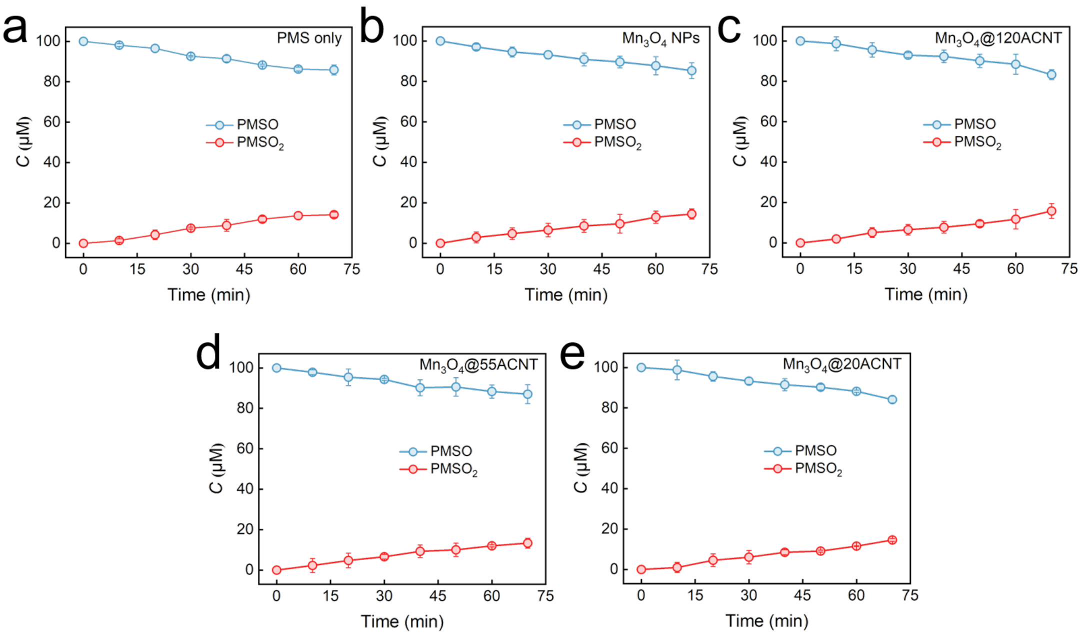

Supplementary Fig. 33 Degradation of PMSO and formation of $PMSO_2$ in different oxidation systems. Conditions: T = 293.2±0.3 K; pH = 7.0±0.1; [PMSO] = 100 μM; [PMS] = 2.0 mM; [catalyst] = 75 mg L^{-1}. The error bars represent the standard deviations from triplicate tests.

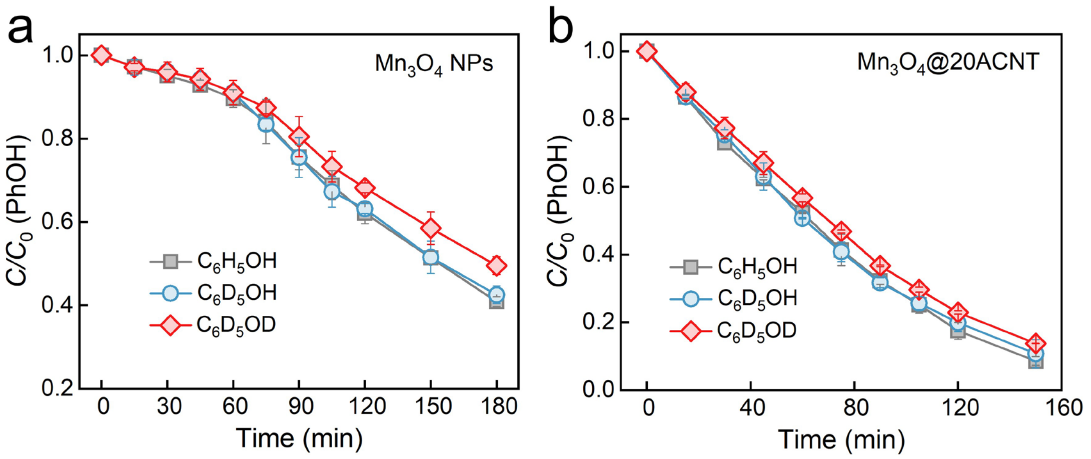

Supplementary Fig. 34 The kinetic isotope effect for PhOH conversion in (a) Mn_3O_4/PMS and (b) Mn_3O_4@20ACNT/PMS. Conditions: T = 293.2±0.3 K; pH = 7.0±0.1; [C_6D_5OD] = [C_6D_5OH] = 200 μM; [PMS] = 2.0 mM; [catalyst] = 75 mg L^{-1}. The error bars represent the standard deviations from triplicate tests.

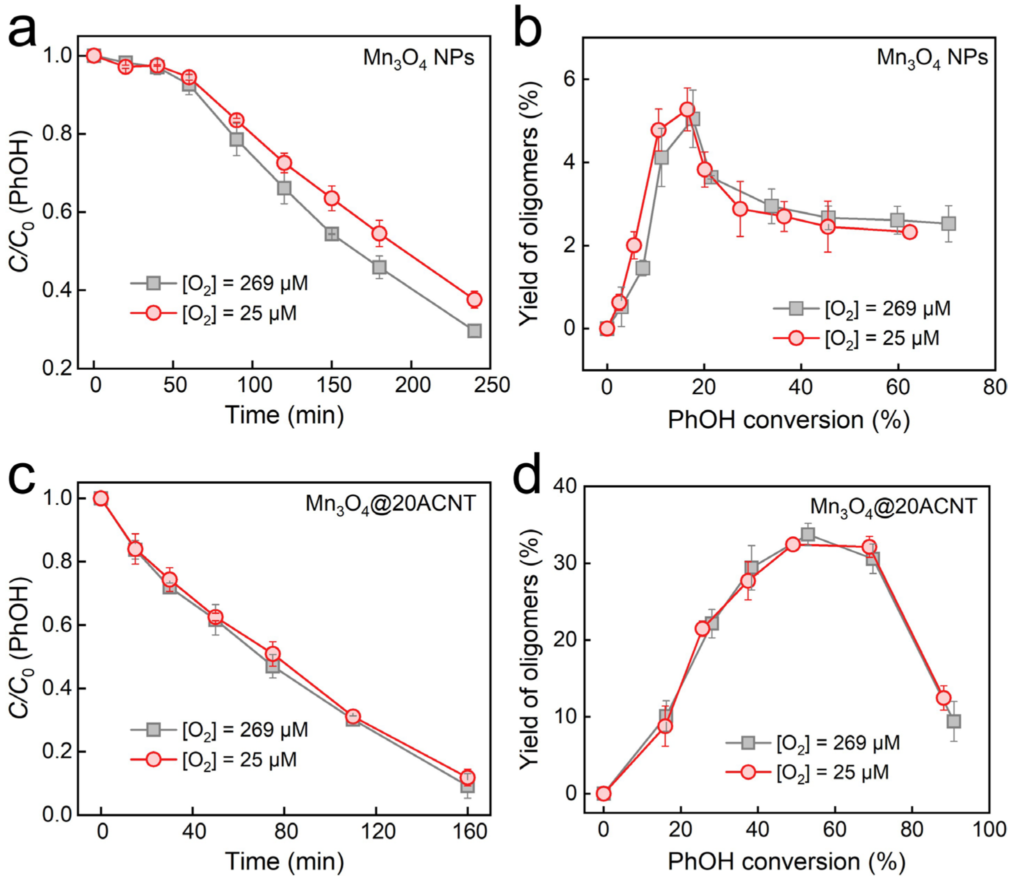

Supplementary Fig. 35 Effect of O_2 concentration on PhOH conversion and oligomer yield in (a–b) Mn_3O_4/PMS and (c–d) Mn_3O_4@20ACNT/PMS. Conditions: T = 293.2±0.3 K; pH = 7.0±0.1; [PhOH] = 200 µM; [PMS] = 2.0 mM; [catalyst] = 75 mg L^{-1}. The error bars represent the standard deviations from triplicate tests.

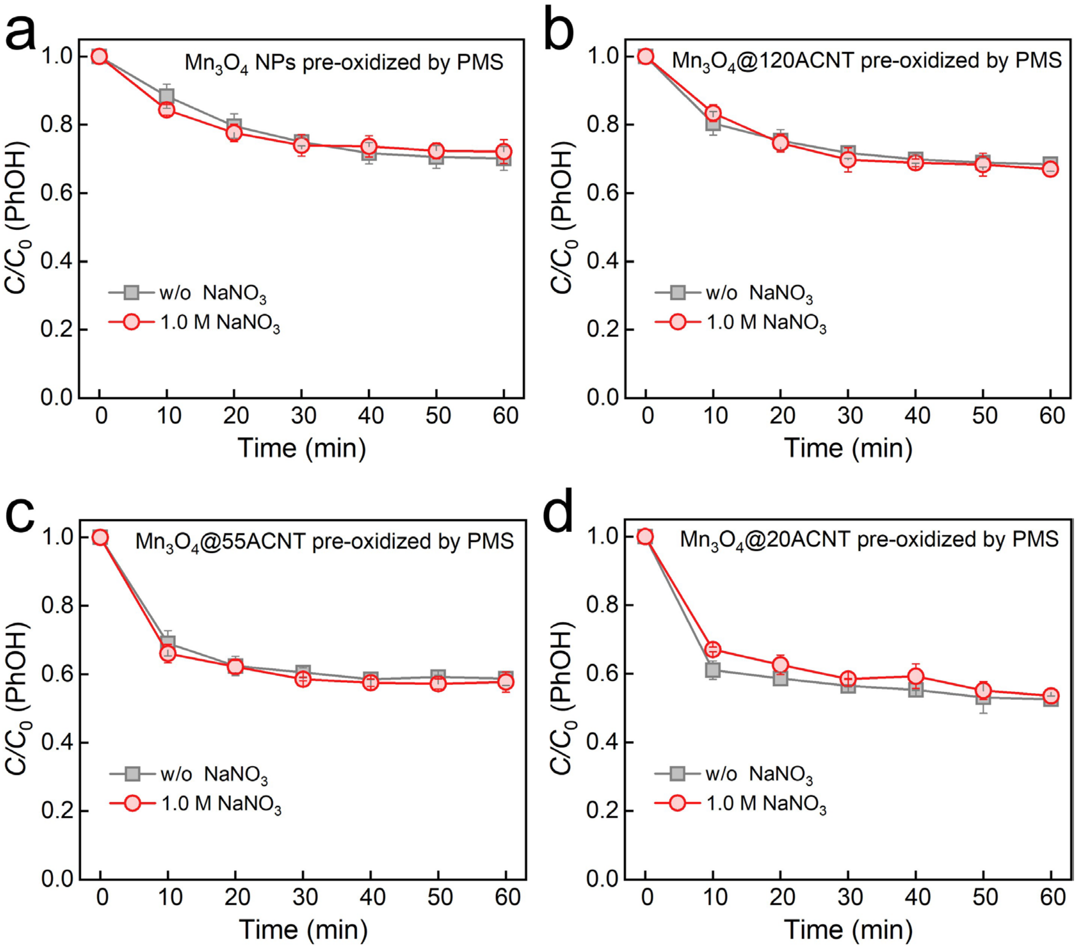

Supplementary Fig. 36 Effect of $NaNO_3$ on PhOH oxidation by pre-oxidized catalysts. Conditions: T = 293.2±0.3 K; pH = 7.0±0.1; [catalyst] = 400 mg L^{-1}; [PhOH] = 50 μM; [PMS] = 10 mM. The error bars represent the standard deviations from triplicate tests.

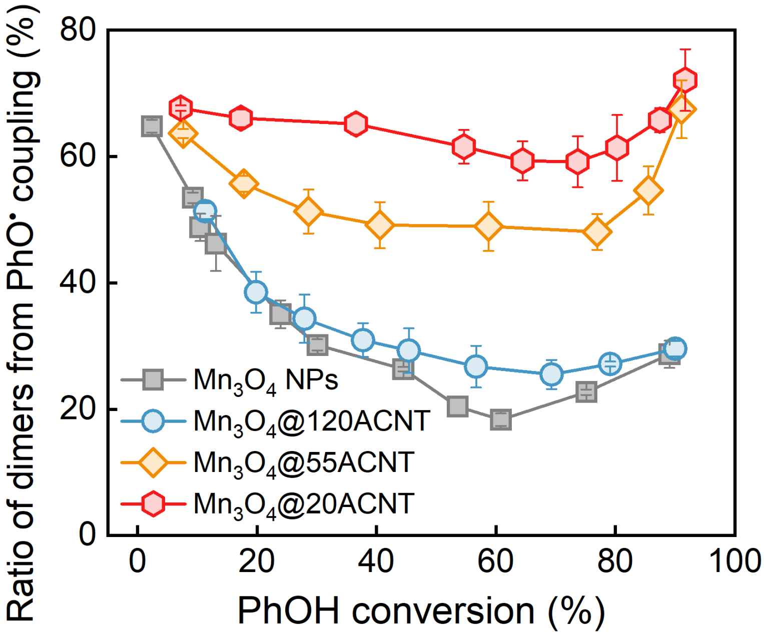

Supplementary Fig. 37 Ratio of the dimers from C–O and C–C coupling of phenoxy radicals in different oxidation systems. The error bars represent the standard deviations from triplicate tests.

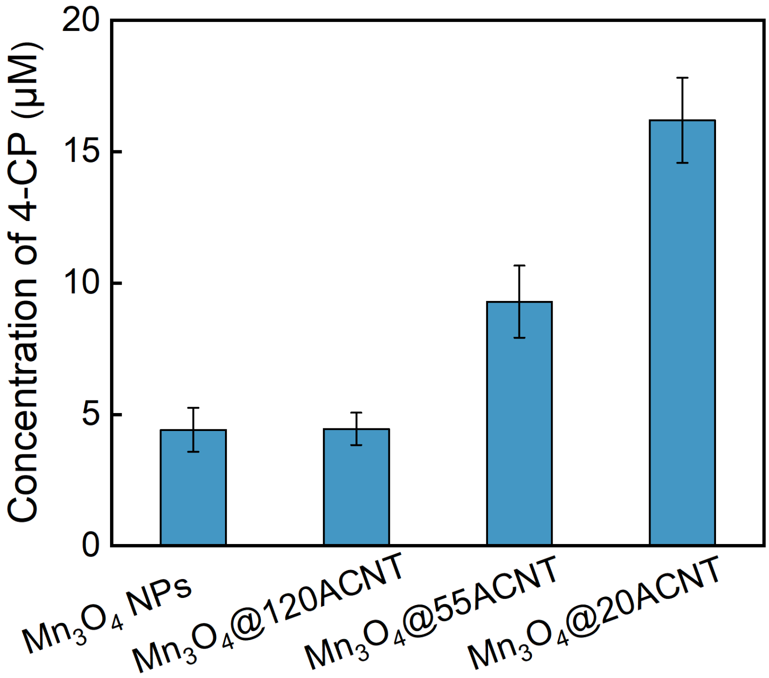

Supplementary Fig. 38 Formation of 4-chlorophenol at 20% PhOH conversion after adding Cl^{-}. Conditions: T = 293.2±0.3 K; pH = 7.0±0.1; [PhOH] = 200 μM; [Cl^{-}] = 50 mM; [PMS] = 2.0 mM; [catalyst] = 75 mg L^{-1}. The error bars represent the standard deviations from triplicate tests.

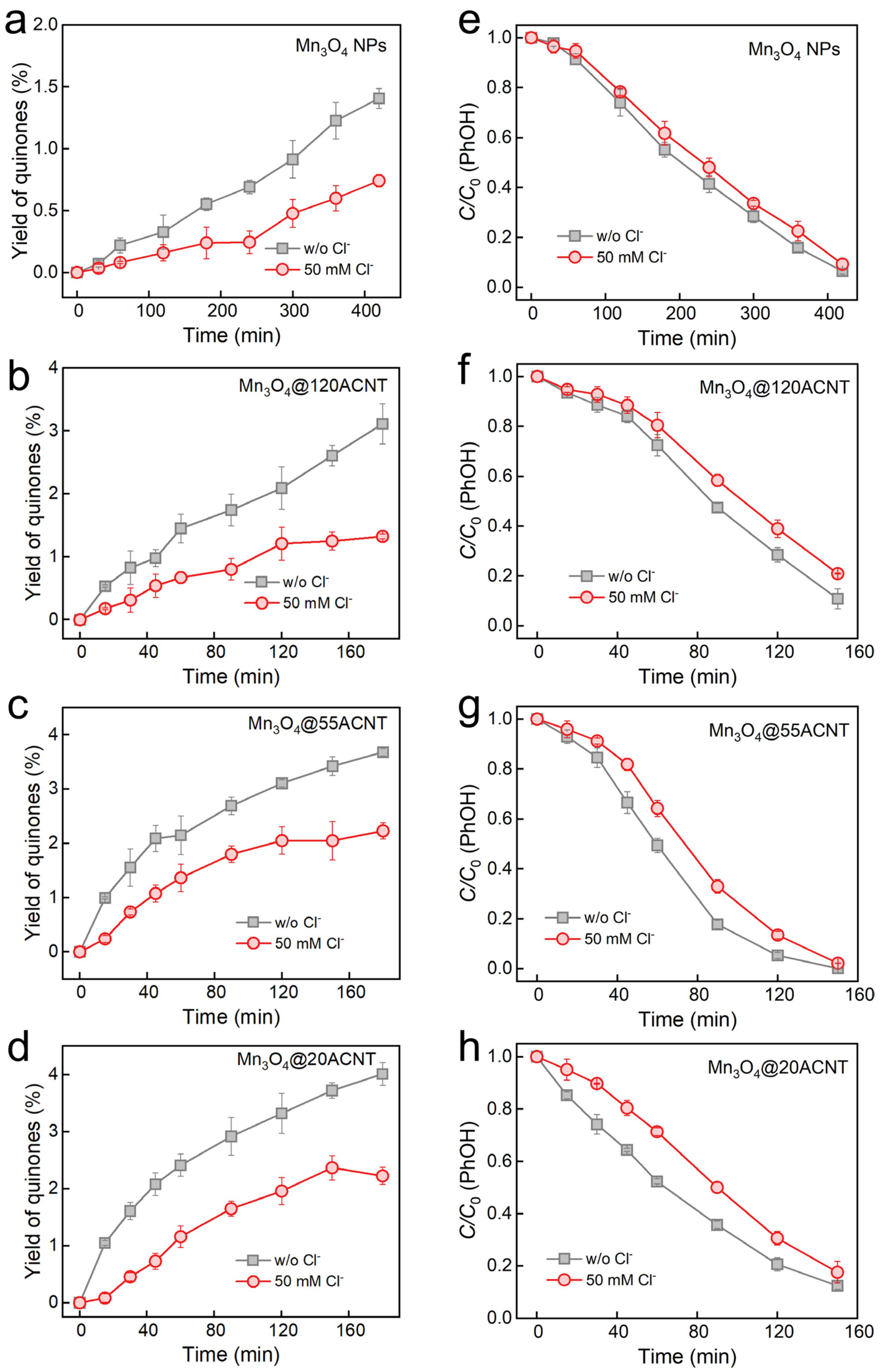

Supplementary Fig. 39 Effect of Cl^- on quinone formation (a–d) and conversion of PhOH (e–h) in different oxidation systems. Conditions: T = 293.2±0.3 K; pH = 7.0±0.1; [PhOH] = 200 μM; [Cl^-] = 50 mM; [PMS] = 2.0 mM; [catalyst] = 75 mg L^{-1}. The error bars represent the standard deviations from triplicate tests.

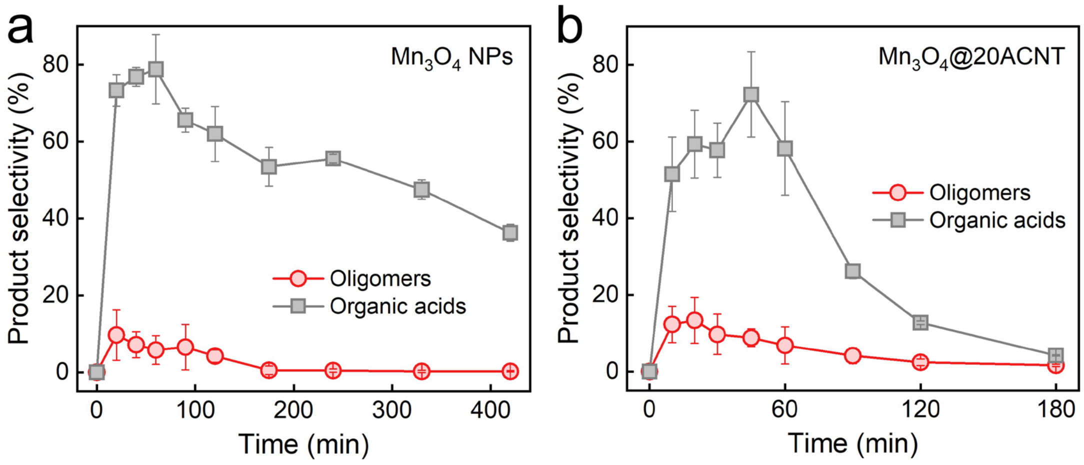

Supplementary Fig. 40 Oxidation of BQ and product selectivity in (a) Mn_3O_4/PMS and (b) Mn_3O_4@20ACNT/PMS. Conditions: T = 293.2±0.3 K; pH = 7.0±0.1; [BQ] = 200 μM; [PMS] = 2.0 mM; [catalyst] = 75 mg L^{-1}. The error bars represent the standard deviations from triplicate tests.

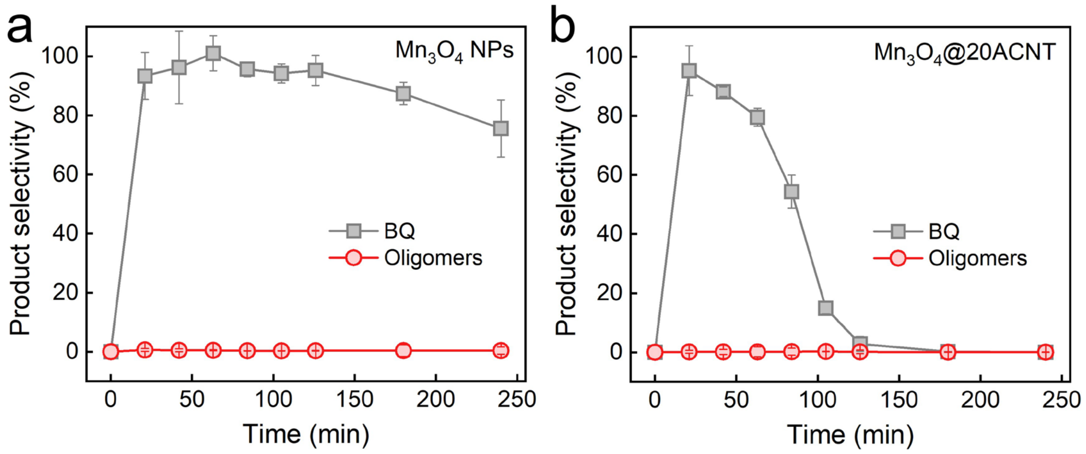

Supplementary Fig. 41 Oxidation of HQ and product selectivity in (a) Mn_3O_4/PMS and (b) Mn_3O_4@20ACNT/PMS. Conditions: T = 293.2±0.3 K; pH = 7.0±0.1; [HQ] = 200 μM; [PMS] = 2.0 mM; [catalyst] = 75 mg L^{-1}. The error bars represent the standard deviations from triplicate tests.

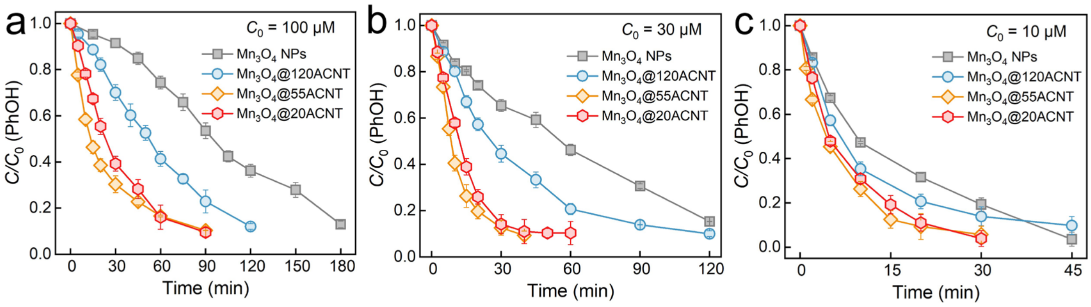

Supplementary Fig. 42 Effect of the concentration of PhOH on the kinetics of PhOH conversion in different catalytic oxidation systems. Conditions: T = 293.2±0.3 K; pH = 7.0±0.1; [PMS] = 2.0 mM; [catalyst] = 75 mg L^{-1}. The error bars represent the standard deviations from triplicate tests.

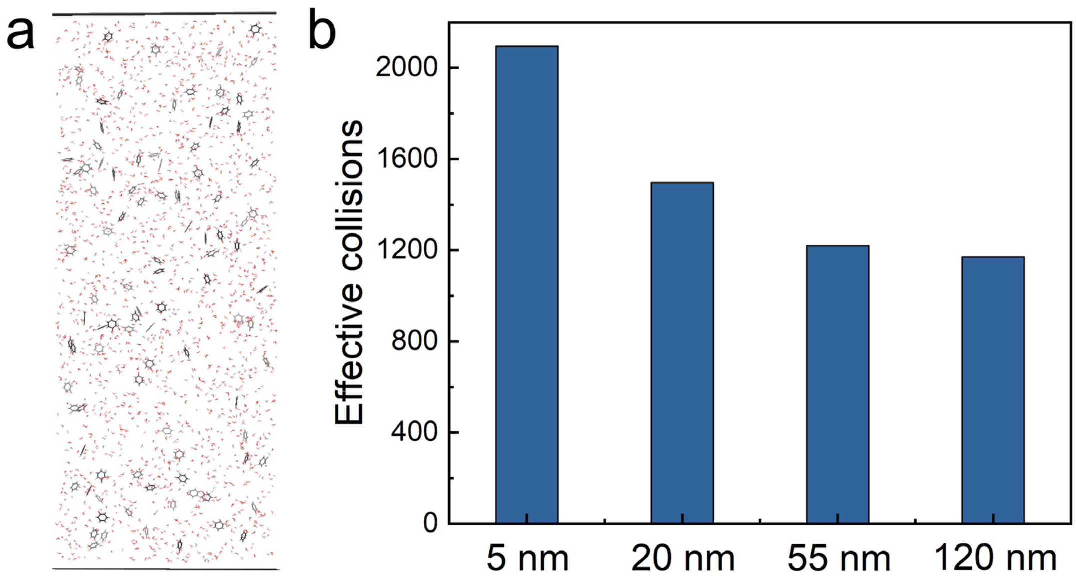

Supplementary Fig. 43 MD simulations of $PhO^{\bullet}$-involved reactions under nanoconfinement with different spatial sizes. (a) A snapshot of the established simulation models. (b) Effective collisions for $PhO^{\bullet}$ coupling in different oxidation systems. The number of H_2O and $PhO^{\bullet}$ were 2000 and 100, respectively. The temperature was set as 2000 K to facilitate collision within a limited interval.

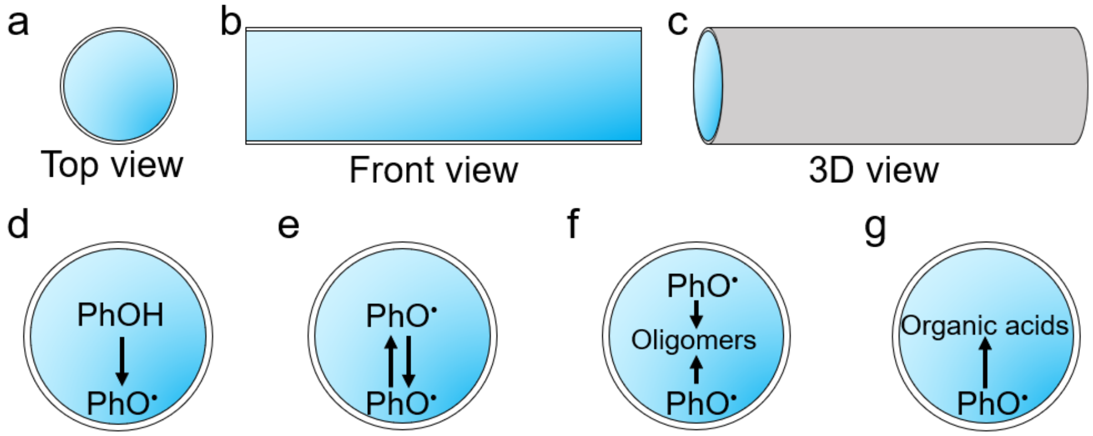

Supplementary Fig. 44 Overview of FEM simulations. (a) Top view, (b) front view, and (c) 3D view of the hollow tubes. (d–g) The chemical reactions: Adsorption of PhOH and subsequent conversion to PhO•; adsorption-desorption equilibrium of PhO•; coupling of adsorbed PhO• with free PhO•; coupling of free PhO•; oxidation of PhO• by the active surface sites to organic acids.

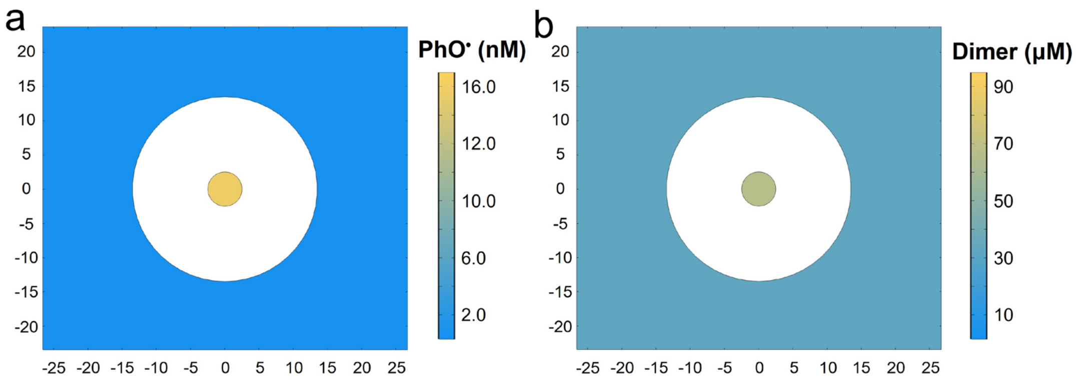

Supplementary Fig. 45 The computed concentration distribution of (a) PhO$^{\bullet}$ and (b) dimer in the mid-section of a 5-nm tube at 50% PhOH conversion. The white circular ring represents the tube wall of 22 nm. The unit of the x and y axis is nm.

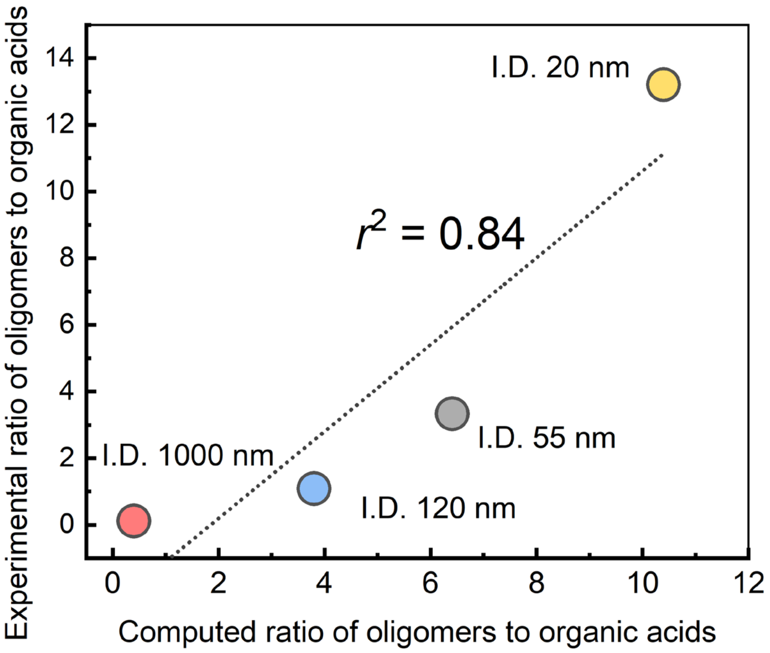

Supplementary Fig. 46 Plots of computed ratio of oligomers to organic acids versus the experimental results. The experimental ratio of oligomers to organic acids is the yield of oligomers to the yield of organic acids at 50% PhOH conversion.

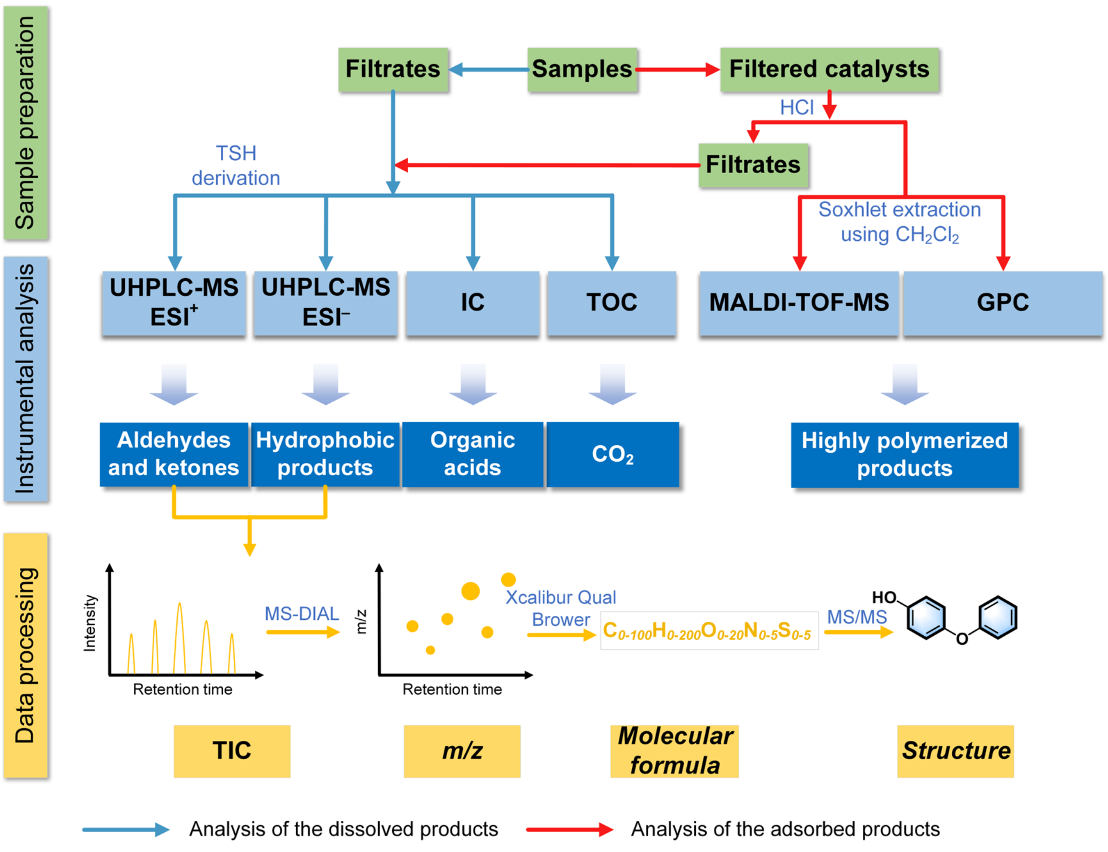

Supplementary Fig. 47 Scheme for the product analysis.

Supplementary References

1 Ochterski, J. W. Thermochemistry in Gaussian. 1-19 (2000).

2 Liang, C., Huang, C.-F., Mohanty, N. & Kurakalva, R. M. A rapid spectrophotometric determination of persulfate anion in ISCO. *Chemosphere* **73**, 1540-1543 (2008).

3 Martínez, L., Andrade, R., Birgin, E. G. & Martínez, J. M. PACKMOL: A package for building initial configurations for molecular dynamics simulations. *J. Comput. Chem.* **30**, 2157-2164 (2009).

4 Thompson, A. P. *et al.* LAMMPS-a flexible simulation tool for particle-based materials modeling at the atomic, meso, and continuum scales. *Comput. Phys. Commun.* **271**, 108171 (2022).

5 Weismiller, M. R., Van Duin, A. C., Lee, J. & Yetter, R. A. ReaxFF reactive force field development and applications for molecular dynamics simulations of ammonia borane dehydrogenation and combustion. *J. Phys. Chem. A* **114**, 5485-5492 (2010).

6 Castro-Marcano, F., Kamat, A. M., Russo Jr, M. F., van Duin, A. C. & Mathews, J. P. Combustion of an Illinois No. 6 coal char simulated using an atomistic char representation and the ReaxFF reactive force field. *Combust. Flame* **159**, 1272-1285 (2012).

7 Vashisth, A., Ashraf, C., Zhang, W., Bakis, C. E. & van Duin, A. C. T. Accelerated ReaxFF simulations for describing the reactive cross-linking of polymers. *J. Phys. Chem. A* **122**, 6633-6642 (2018).

8 Rapp, B. E. *Microfluidics: modeling, mechanics and mathematics*. (Elsevier, 2022).

9 Poling, B. E. *The properties of gases and liquids*. (2004).

10 Li, Y. *et al.* Transplacental transfer of per-and polyfluoroalkyl substances identified in paired maternal and cord sera using suspect and nontarget screening. *Environ. Sci. Technol.* **54**, 3407-3416 (2020).

11 Wang, X. *et al.* Suspect and non-target screening of pesticides and pharmaceuticals transformation products in wastewater using QTOF-MS. *Environ. Int.* **137**, 105599 (2020).

12 Yu, N. *et al.* Nontarget discovery of per-and polyfluoroalkyl substances in atmospheric particulate matter and gaseous phase using cryogenic air sampler. *Environ. Sci. Technol.* **54**, 3103-3113 (2020).
